# Can you *feel* what I am saying? Speech-based vibrotactile stimulation enhances the cortical tracking of attended speech in a multi-talker background

**DOI:** 10.1162/IMAG.a.1305

**Published:** 2026-07-21

**Authors:** I. Sabina Răutu, Mathieu Bourguignon, Vincent Wens, Veikko Jousmäki, Julie Bertels, Xavier De Tiège

**Affiliations:** Laboratoire de Neuroanatomie et Neuroimagerie translationnelles (LN^2^T), UNI – ULB Neuroscience Institute, Université libre de Bruxelles (ULB), Brussels, Belgium; Laboratory of Functional Anatomy, Université libre de Bruxelles (ULB), Brussels, Belgium; WEL Research Institute, Wavre, Belgium; Department of Translational Neuroimaging, CUB Hôpital Erasme, Hôpital Universitaire de Bruxelles (H.U.B.), Université libre de Bruxelles (ULB), Brussels, Belgium; Aalto Neuroimaging, Aalto University, Espoo, Finland; ULBabylab, Center for Research in Cognition and Neurosciences (CRCN), UNI – ULB Neuroscience Institute, Université Libre de Bruxelles (ULB), Brussels, Belgium

**Keywords:** cortical speech tracking, audio-tactile processing, speech-in-noise perception, multi-talker background, magnetoencephalography (MEG), speech-brain coherence

## Abstract

In environments with multiple talkers, humans can ‘tune in’ to a speaker of interest while ignoring competing voices. In such conditions, however, auditory cortices track the attended speech envelope rhythms (cortical tracking of speech, CTS) less accurately than in quiet, hindering intelligibility. Visual speech cues (e.g., lip movements) can enhance this CTS, but it remains unclear whether other non-auditory sensory cues, such as tactile input, provide comparable benefits through similar neural mechanisms. Here, using magnetoencephalography, we quantified CTS as the coherence between the speech temporal envelope of the attended speaker and brain responses in supratemporal auditory areas at syllabic (4–8 Hz), word (1–4 Hz) and phrasal/sentential (<1 Hz) frequencies. Participants listened to connected speech presented alone, together with synchronous or asynchronous speech-based vibrations, or with the corresponding speaker video, in both quiet and a multi-talker background. We hypothesized that, in the presence of competing background speakers, speech-based vibrotactile stimulation improves comprehension by enhancing CTS of the attended speaker and modulating auditory-seeded functional brain connectivity with extra-auditory neocortical brain areas. Results revealed that synchronous vibrotactile stimulation improved comprehension in the multi-talker background and increased syllabic CTS at the right auditory cortex, with this CTS increase magnitude correlating with comprehension performance. Audio-tactile CTS enhancement was accompanied by stronger beta-band auditory cortex connectivity with ipsilateral angular and ventral inferior temporal gyri, alongside reduced alpha-band coupling with the precuneus. These findings suggest that vibrotactile input can support speech-in-noise processing by impacting both local auditory cortical activity and auditory-seeded long-range functional connectivity.

## 1 Introduction

Noisy social environments with simultaneous talkers, such as crowded classrooms or busy cafés, create challenging acoustic conditions for speech comprehension. When faced with this scenario, listeners often engage demanding high-level perceptual and cognitive processes (Bronkhorst, 2015; Zion Golumbic et al., 2013b) to effectively attend to the speaker of interest and tune out competing talkers. Visual speech cues (e.g., articulatory facial gestures) can reinforce the perceptual segregation of the attended speaker from such a multi-talker background (Schwartz et al., 2004; Stacey et al., 2016; Sumby & Pollack, 1954; Zion Golumbic et al., 2013a), improving intelligibility. Intriguingly, tactile stimulation conveying speech-derived cues can also robustly bolster speech-in-noise recognition, including in multi-talker backgrounds, of both hearing-impaired and normal-hearing listeners (Cieśla et al., 2019; Fletcher et al., 2018; Huang et al., 2017; Răutu et al., 2023; Schulte et al., 2023). Still, the precise neural substrates underlying audio-tactile enhancement in multi-talker backgrounds remain to be fully elucidated.

When exposed to connected speech, auditory cortical rhythms synchronize with (i.e., track) the slow (<8 Hz), quasi-rhythmic amplitude fluctuations of the speech temporal envelope (Bourguignon et al., 2013; Gross et al., 2013; Lalor & Foxe, 2010; Luo & Poeppel, 2007; Poeppel & Assaneo, 2020; Rosen et al., 1997; Zoefel, 2018). This cortical tracking of speech (CTS) has been observed at timescales matching syllabic (4–8 Hz), word (1–4 Hz) and phrasal/sentential (<1 Hz) rates (Bourguignon et al., 2013; Keitel et al., 2018; Luo et al., 2010; Luo & Poeppel, 2007; Vander Ghinst et al., 2016). CTS is thought to underlie speech recognition by supporting the parsing of continuous speech input into its respective hierarchical linguistic constituents (Ding & Simon, 2014; Giraud & Poeppel, 2012) and, crucially, has been linked to speech intelligibility and comprehension (Keitel et al., 2018; Molinaro & Lizarazu, 2018; Peelle et al., 2013; Peelle & Davis, 2012; Riecke et al., 2018; Vander Ghinst et al., 2016). In multi-talker backgrounds, CTS is selective for the attended speaker’s temporal envelope rather than those of competing background speakers (Ding & Simon, 2012; Puvvada & Simon, 2017; Zion Golumbic et al., 2013b). Yet, it is markedly reduced relative to quiet (Vander Ghinst et al., 2016, 2019). Multi-talker listening, thus, provides an ecologically relevant setting allowing for the assessment of how non-auditory, speech-relevant sensory cues may counteract the degradation of the attended speaker’s CTS.

In auditory settings with one (Zion Golumbic et al., 2013a) and multiple competing background talkers (Bertels et al., 2023; Destoky et al., 2020), visual speech cues of the attended speaker can effectively increase CTS, providing a potential mechanistic foundation for the behavioural benefits of visual speech in multi-talker backgrounds. This effect has been attributed to the capacity of visual speech to elicit neural tracking in auditory cortices (Aller et al., 2022; Bourguignon et al., 2020), as well as provide temporally-relevant information which may support speech stream selection and parsing through attentional processes (Zion Golumbic et al., 2013b). Evidence of speech comprehension enhancement in multi-talker backgrounds by speech-derived tactile stimulation (Cieśla et al., 2019; Răutu et al., 2023) suggests that this cross-modal CTS support may generalize to the somatosensory modality. This perspective is supported by evidence showing that vibrotactile input can shape auditory processes through a direct modulation of activity in auditory cortices (Caetano & Jousmäki, 2006; Fu et al., 2003; Hackett et al., 2007; Kim et al., 2020; Lakatos et al., 2007; Murray et al., 2005; Shen et al., 2018), including hemodynamic responses to audio-tactile speech (Schulte et al., 2025). Vibrotactile stimulation transmitting speech-related cues has even been shown to enhance CTS of degraded speech in quiet (Riecke et al., 2019) and of speech embedded in spectrally-matched noise (Guilleminot & Reichenbach, 2022). Several caveats, however, limit the interpretation of these findings with respect to audio-tactile (and, more broadly, multisensory) effects on CTS in multi-talker backgrounds.

First, audio-tactile CTS benefits may not emerge in the ecologically valid context of actively segregating a target speaker’s connected speech stream from competing ones. CTS is more strongly attenuated by a multi-talker background than by non-speech noise (Destoky et al., 2020), which may relate to differences in the nature of the interference introduced by these maskers. Vocoding and energetic (non-speech) noise primarily degrade peripheral audibility (Ihlefeld & Shinn-Cunningham, 2008), while multi-talker backgrounds introduce additional informational interference beyond energetic overlap (Brungart et al., 2001). This may impede auditory object formation, necessitating the engagement of top-down, central selection processes (Rimmele et al., 2015; Zion Golumbic et al., 2013b). Whether a speech-derived vibrotactile signal can support this segregation and improve the CTS of the attended speaker’s envelope is, to the best of our knowledge, yet to be confirmed. Moreover, in terms of anatomical specificity, the use of electroencephalography and absence of source reconstruction limit the spatial precision with which previous audio-tactile CTS effects were attributed to auditory cortices (Guilleminot & Reichenbach, 2022; Riecke et al., 2019). Cross-modal, audio-tactile CTS improvements observed in the auditory cortex may involve primary, secondary, or other cortical areas along the superior temporal sulcus. They can also be accompanied by top-down influences from high-order, associative cortical areas through functional connectivity changes. Such top-down mechanisms have been demonstrated for audio-visual speech perception (Giordano et al., 2017), but remain unsettled for audio-tactile speech, despite evidence of extra-auditory functional connectivity changes following audio-tactile speech training (Cieśla et al., 2025). Thus, uncovering the distinct contributions of auditory and extra-auditory activity to audio-tactile CTS enhancement requires further investigation. Lastly, prior audio-tactile work has quantified CTS using backward (decoding) stimulus reconstruction (Riecke et al., 2019) and forward (encoding) temporal response function modeling (Guilleminot & Reichenbach, 2022). These approaches model CTS as a time-lagged stimulus-response relationship, either by predicting neural activity from speech features or by reconstructing speech features from neural activity (Crosse et al., 2016). In contrast, coherence (Halliday et al., 1995) quantifies the frequency-specific coupling between speech and neural activity, providing a measure of rhythmic synchronization rather than an explicit speech-brain temporal mapping (Molinaro, 2025). The frequency-specific nature of coherence makes it particularly suited to examine the impact of speech-derived vibrotactile stimulation across syllabic, word, and phrasal/sentential timescales. To our knowledge, audio-visual and audio-tactile effects on CTS have also not been compared intra-individually using this approach. Consequently, whether multisensory (tactile or visual) benefits in multi-talker listening are accompanied by strengthened frequency-specific CTS in the auditory cortex, and how comparable or distinct these effects are across modalities within individuals, remains unresolved.

**Fig. 1.**
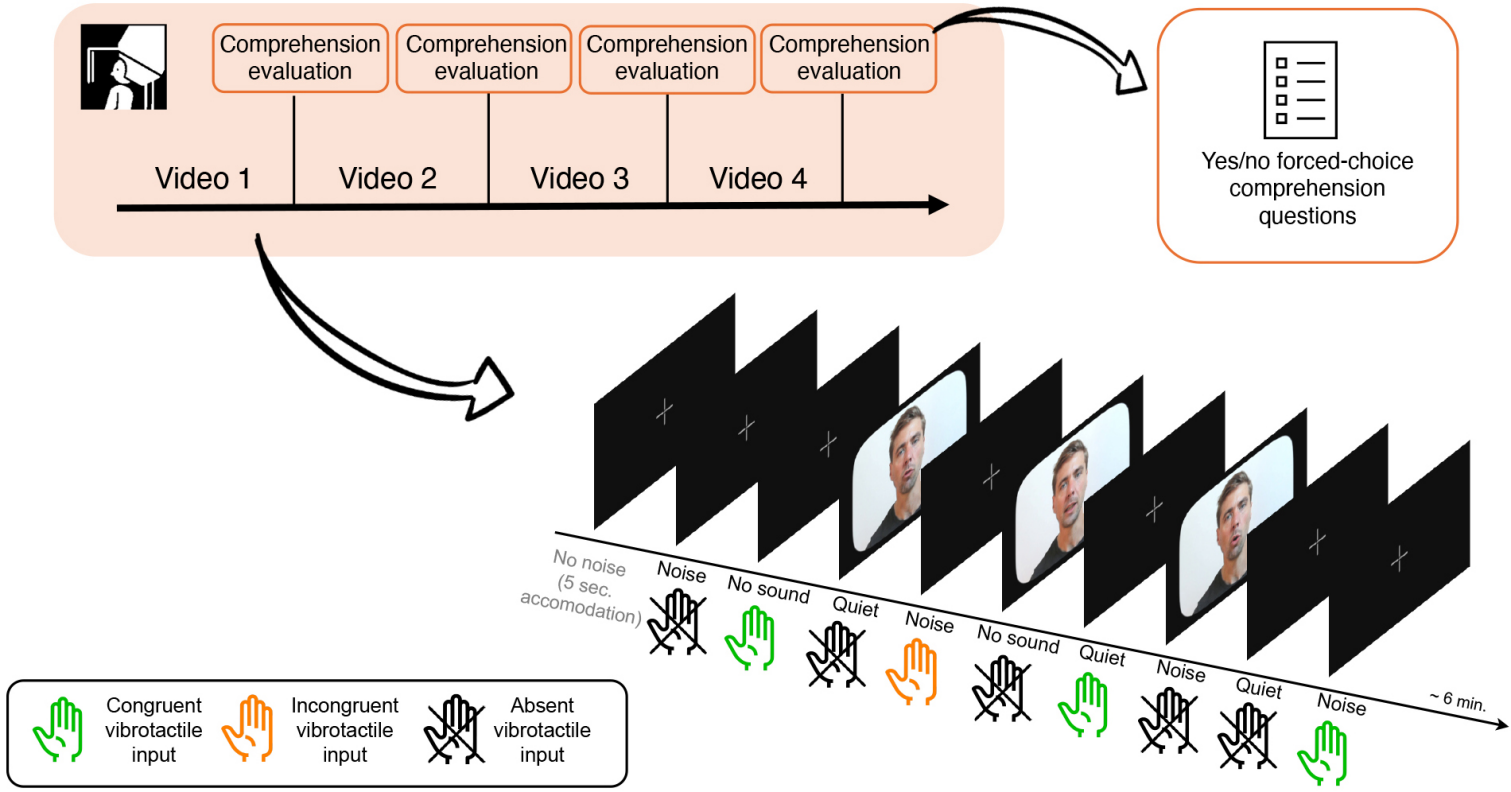
Illustration of the experiment progression during the MEG acquisition (orange panel) and a time-course of a video stimulus. Participants viewed four videos in total, each segmented into 9 blocks corresponding to the experimental conditions (mean block duration: ~40 seconds per video). In audio-visual conditions, the visual input was always presented in congruence with the auditory speech signal.

In the present study, we aimed to address the above-mentioned gaps and, more broadly, investigate whether CTS in auditory cortices, as measured using the coherence between attended speech and brain signals, is a core mechanism associated with audio-tactile speech benefits in a multi-talker background. We analyzed magnetoencephalographic (MEG) recordings acquired from 30 normal-hearing adults while they watched four videos containing naturalistic spoken narratives (~6 minutes each; Fig 1). CTS was quantified as the coherence between the attended speech envelope and MEG signals at syllabic (4–8 Hz), word (1–4 Hz), and phrasal/sentential (<1 Hz) timescales. For increased anatomical specificity, coherence estimates were source-projected and analyses focused on CTS obtained at bilateral auditory cortices. Speech was delivered either in quiet or mixed with a multi-talker background of equal intensity (i.e., signal-to-noise ratio (SNR): 0 dB). Three distinct sensory conditions were presented: unimodal (auditory-only, A-only), speech paired with a unimanual (left hand) speech-derived vibrotactile stimulation (audio-tactile, AT) previously shown to improve speech recognition in multi-talker backgrounds (Răutu et al., 2023), or speech accompanied by a video of the speaker’s face while speaking (audio-visual, AV; in non-AV conditions a fixation cross was displayed). This design allowed us to additionally investigate if AT benefits rely on similar CTS-based mechanisms as in AV conditions. To determine whether temporal alignment is obligatory for audio-tactile CTS enhancement, we also manipulated the vibrations’ temporal congruency, contrasting multi-talker conditions in which they were aligned (congruent AT condition; ATc) or misaligned (incongruent AT condition; ATi) with the attended speech envelope. To further assess how non-auditory modality-specific cues shape CTS independently from the auditory input and potential super-additive effects, we also included visual-only (V-only) and tactile-only (T-only) conditions. Critically, we established a direct link between neural-level changes in CTS and behavior by employing condition-specific assessments of comprehension using content questions. Finally, we examined the functional connectivity seeded from auditory cortices involved in CTS in the multi-talker ATc condition.

## 2 Materials and Methods

### 2.1 Participants

Thirty native French-speaking adults (15 female) aged 25.5 ± 5.0 years (mean ± s.d.) participated in this study. All participants were right-handed based on the Edinburgh Handedness Inventory (Oldfield, 1971). All had normal hearing as indicated by hearing thresholds (air-conducted) of ≤25 dB HL (hearing level) obtained during pure-tone audiometric testing at all standard frequencies between 125 and 8000 Hz. Participants’ central auditory perception was also evaluated using a standardized French language battery (Demanez et al., 2003) and a speech-in-noise recognition threshold measurement using the French Sentence Test for Speech Intelligibility in Noise (FIST) (Luts et al., 2008; procedure described in Răutu et al., 2023), with no participants demonstrating atypical auditory abilities. They all had normal or corrected-to-normal vision and normal somatosensory perception, with the latter being confirmed through a vibrotactile threshold assessment (Răutu et al., 2023). Participants completed a brief questionnaire to report their current medication or history of psychiatric, neurological, auditory, or somatosensory disorders. No relevant pathology was indicated. The study was approved by the ULB—Hôpital Erasme Ethical Committee (P2012/049). Written informed consent was obtained from all participants prior to testing and they received monetary compensation for their participation.

### 2.2 Stimuli

Stimuli were derived from eight audio-visual recordings of trained, native French-speaking narrators (one male and one female, four recordings per narrator) speaking for ~6 minutes (6.0 ± 0.3 minutes). During video recording, speakers were instructed to speak with little to no facial expressions and head or body movements. The linguistic content of the recordings included the characteristics and habitat of eight uncommon animals: the axolotl, tardigrade, dugong, kiwi, wombat, Dumbo octopus, Trionyx, and aye-aye. This specific topic was chosen for its emotional neutrality (Bertels et al., 2014), richness in informational content, and, given the animals’ rarity, the difficulty to intuit information during the comprehension evaluation. This was confirmed *a posteriori* following the MEG data recording, as no participant indicated in-depth familiarity with the heard information. Speech content comprehension was assessed using yes/no forced-choice questions, targeting condition-specific informational content present only in the attended speech stream. Given the long (>5 minutes), naturalistic nature of the connected speech stimuli, this ensured that participants engaged with the narrative continuously, without disruptive task switching (e.g., target-word detection or button press responses). Videos were first edited using Final Cut Pro (v10.6.5, Apple Inc.) to remove prolonged pauses and errors. Recordings were slowed down as needed to ensure clear comprehension and consistent syllabic rhythm across recordings (final mean syllabic rate: 5.61 ± 0.28 Hz). Based on the recordings, experimental stimuli were prepared as described below.

#### 2.2.1 Auditory stimuli

Video soundtracks were extracted, denoised, and intensity-normalized across videos (root mean square procedure) using the Audacity audio editing software (v3.0.2; https://www.audacityteam.org). For the multi-talker conditions, an auditory background composed of 6 native French speakers (3 females) talking simultaneously (Perrin & Grimault, 2005) was normalized and mixed with the soundtracks at 0 dB SNR using custom Matlab (R2021a, TheMathWorks) scripts. An SNR of 0 dB was chosen to maintain moderate intelligibility levels and for its reported ability to significantly reduce CTS in multi-talker backgrounds (Brungart et al., 2001; Vander Ghinst et al., 2019).

#### 2.2.2 Tactile stimuli

**Fig. 2.**
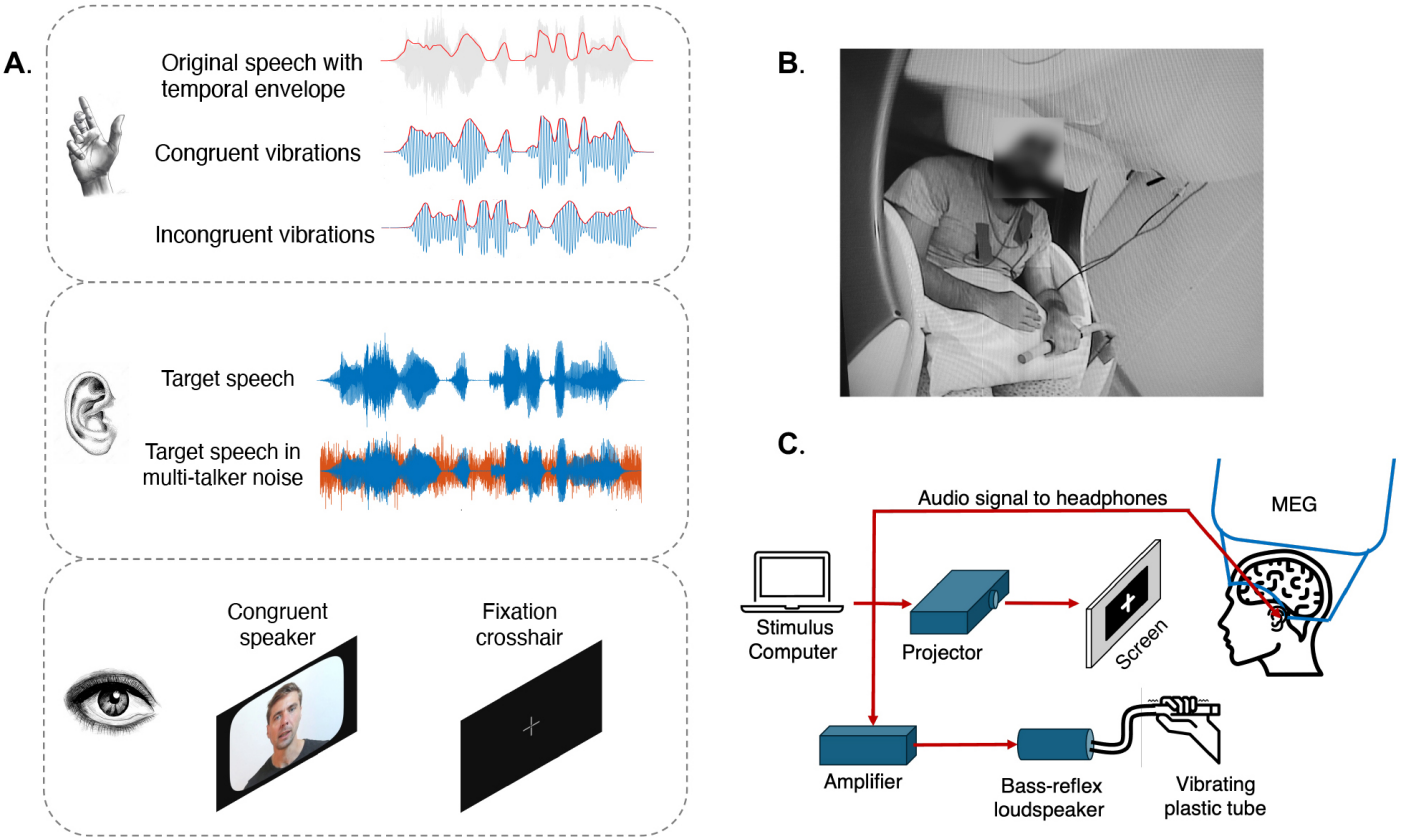
Experimental setup and material. (A) Vibrotactile inputs (palm symbol) were derived from the speech envelope of the heard speech signal (first row, in red); congruent vibrations (middle row) had amplitude variations (in red) as per the speech temporal envelope, while incongruent vibrations (bottom row) also had envelope-based amplitude variations (in red) but not aligned with the heard speech. Auditory (ear symbol) inputs consisted of clear speech or speech mixed with a multi-talker background (in orange). Visual (eye symbol) information was either the speaker talking or a fixation cross. (B) A participant holding the vibrating tube while seated in the MEG chair. (C) A main stimulus computer was used to simultaneously deliver the vibrotactile, video, and auditory stimulation. Auditory signals were transmitted through MEG-compatible earphones. Visual stimuli were projected on an MEG-compatible screen through a dedicated projector. Vibrotactile signals reached participants’ palms through a plastic tube with a vibrating silicone ending.

Tactile stimuli consisted of vibrations derived from the speech temporal envelope of the unaltered (i.e., unmixed with the multi-talker background) speech signal of each video. The generation of the vibrations was done using custom Matlab (R2021a, TheMathWorks) scripts (Răutu et al., 2023). First, for each normalized clean audio track from the original 8 videos, the corresponding speech envelopes were extracted using an established approach (Bertels et al., 2023) which entailed passing each audio track through a 31-channel gammatone filter bank ranging from 150 to 7000 Hz (Mel scale), followed by computing the temporal envelope of each channel using the Hilbert transform. Then, all channel-specific envelopes were averaged to obtain a composite speech temporal envelope. The gammatone filter frequency interval was chosen to overlap with both the average speech spectrum and the frequency range of human auditory perception (Merchel & Altinsoy, 2020). Figure 2A illustrates the envelope obtained from an excerpt of one audio segment. Finally, the computed temporal envelope was used to amplitude modulate a 150 Hz sinusoidal carrier, the prime sensitivity range of the principal vibratory mechanoreceptors, Pacinian corpuscles (Gandhi et al., 2011).

#### 2.2.3 Visual stimuli

In AV conditions, the visual component of the experimental videos was the speaker’s face while speaking; for all other conditions, a central grey cross was presented against a black background. Minimal editing was applied to the speaker recordings, comprising only color and luminance equilibration across videos, frame centering, and the addition of a black border (all editing done in Final Cut Pro, Apple Inc.).

#### 2.2.4 Final video generation

Final video editing and condition assignment were done using custom Python scripts (v3.9.5, Python Software Foundation, https://www.python.org/). Each recording was divided into nine equal blocks assigned to nine experimental conditions. Of these, seven blocks included sound and two were soundless (i.e., V-only and T-only). Blocks with auditory content were either unimodal (A-only) or bimodal; for the latter, the auditory input was paired with either visual (i.e., AV) or tactile (i.e., AT) input. For each sensory input type (A, AV, or AT), two blocks were presented: one in which the attended speech was presented in quiet, and the other in which it was embedded in multi-talker background, totaling six conditions. For the four bimodal conditions among these six, the visual/tactile signal was always synchronous with the attended speech stream. To assess the effect of vibration congruency, a seventh multi-talker condition presented vibrations derived from the speech envelope of a distinct audio segment from the same recording (i.e., ATi; Fig. 2A). Soundless conditions did not contain any auditory input but presented either the original video of the speaker’s face (V-only) or congruent vibrations derived from the unheard auditory input (T-only).

Figure 1 illustrates an example of a video used during the experiment. The first 5 seconds of each video featured the original audio of the attended speaker, enabling participants to identify the narrator’s voice. Condition-to-block assignment was pseudo-randomized, with the first condition always containing sound (for continuity with the initial 5 seconds of the narrator voice), multi-talker conditions always alternating with either quiet or soundless conditions, and successive conditions always presenting differing sensory input types (for example, two AT conditions never occurred consecutively). Based on these constraints, four predefined condition orders were established (see Supplementary File S1) and used to generate four distinct versions of each of the original eight recordings.

### 2.3 Procedure

The experimental stimuli presentation and neural data recording took place in a quiet, magnetically shielded room (De Tiège et al., 2008). Participants were seated comfortably in the MEG chair with their head inside the MEG helmet. Their arms were placed in front of them, and they were asked to hold with their left hand a vibrotactile stimulation tube, without exerting force (Fig. 2B). The left hand was preferred over the right as stronger cortical facilitation effects in primary and secondary contralateral somatosensory cortex have been linked to left-handed vibrotactile stimulation in right-handers (Jin et al., 2020). Moreover, no significant left-right hand difference has been found in the effects of vibrotactile stimulation on speech-in-noise understanding (Răutu et al., 2023). Participants were instructed to listen to the narrator’s voice as attentively as possible even in the presence of any background noise, to watch the videos when presented (otherwise to focus on the fixation cross), to maintain contact with the vibrating tube continuously throughout the video, and to move as little as possible.

Four videos were presented to each participant, resulting in a data length of ~2.5 minutes per condition. Each of the four presented videos had one of the four predefined condition orders, with no order repetition, and the narrator gender was alternated across videos (two males, two females). Video-order assignment was counterbalanced across participants, and we monitored the frequency of the presentation order of the video (e.g., how often a specific video was shown first) and the frequency with which each predefined condition order was assigned to each video position (e.g., how frequently the video presented first had conditions in the first predefined order).

After each MEG recording (one per watched video), participants answered nine yes/no comprehension questions corresponding to the nine experimental conditions from the video. Questions were displayed on the screen, with participants responding verbally through a microphone positioned near the head. Responses (yes/no) were encoded by the experimenter in real time. Following the videos, a 5-minute resting-state MEG recording was acquired (eyes open, no audio, fixation cross displayed).

### 2.4 Hardware

The experimental setup is illustrated in Figure 2C. Recordings were played from the stimuli presentation computer using VLC media player (v3.0.12, VideoLAN Project, GNU General Public License). Videos were displayed on a back-projection screen placed ~1 m in front of the participants. Auditory stimuli were transmitted diotically via MEG-compatible earphones (TIP 300, Natus Inc., Wisconsin, USA) with noise-reducing inserts (ER3-14A, Etymotic Inc., IL, USA), with an average sound intensity of ~65 dB SPL (sound pressure level) as assessed at the earphone level with a sound level meter (Sphynx Audio System). The vibrations were transmitted using an MEG-compatible vibrotactile stimulator similar to the one used in prior studies on the vibrotactile modulation of auditory cortical activity (Caetano & Jousmäki, 2006) and other works (Răutu et al., 2023). Vibrotactile input was sent from the experimental computer to an amplifier connected to a bass-reflex loudspeaker, which then delivered the vibrations through a blind-ended rigid plastic tube (⌀38 mm, 3-m long) partially fixed laterally to the participants such that it could be held passively. The ending of the plastic tube was made from a flexible, vibratory-purposed silicone that had sound-attenuating material (cotton wool) at the closed end, which limited the vibrating tube area to a visually marked palm-sized region. Participants were instructed to respect the limits of these markings while in contact with the tube (Fig. 2B). Amplifier settings were kept constant throughout the experiment, ensuring a fixed vibration intensity across participants. The vibration magnitude was chosen to elicit a clear sensation of vibration during stimulation (as evaluated during behavioural piloting, *n* = 5), without the concomitant emergence of any auditory percept. This intensity was reduced by approximately 10 dB during vibrotactile threshold measurements.

### 2.5 MEG data acquisition

MEG signals were recorded (sampling rate, 1 kHz; band-pass filter, 0.1–330 Hz) at the HUB—Hôpital Erasme using a whole-head MEG system (Triux, MEGIN Oy, Espoo, Finland). The sensor array of the MEG system comprised 306 sensors grouped in 102 triplets of one magnetometer and two orthogonal planar gradiometers. The MEG system was placed in a lightweight, magnetically shielded room (De Tiège et al., 2008). The audio presented to the participants was simultaneously recorded (sampling rate, 1 KHz; low-pass, 330 Hz) with the MEG signals to synchronize MEG and audio signals during the data analysis. To continuously monitor the participants’ head position within the MEG helmet, four head position indicator (HPI) coils were attached to their scalp prior to the start of the MEG session. The position of the coils with respect to three anatomical fiducials (nasion and the left/right preauricular points), as well as to at least 300 points on the surface of the head were digitized with an electromagnetic tracker (FASTRAK, Polhemus, Colchester, USA). Following MEG, participants underwent a high-resolution 3D cerebral magnetic resonance imaging (MRI) T1-weighted scan in a hybrid 3T PET-MR scanner (SIGNA, GE Healthcare, Wisconsin, USA).

### 2.6 MEG data analysis

#### 2.6.1 Preprocessing

First, to suppress external sources of magnetic interference and correct for any head movement, MEG data were preprocessed off-line using the temporal signal space separation (tSSS) algorithm implemented in MaxFilter (Neuromag, Elekta; correlation limit 0.9, segment length 10 seconds) (Taulu et al., 2005; Taulu & Simola, 2006). Then, to eliminate physiological artifacts, independent component analysis (Vigario et al., 2000) using the FastICA algorithm was ran and 30 independent components were extracted from the low-pass filtered MEG data (filter cutoff: 25 Hz). The obtained independent components were visually inspected and, based on their time-course and topography, those corresponding to heartbeat, eyeblink, and eye movement-related artifacts were identified and subtracted from the full-rank MEG signals. MEG data segments corresponding to each experimental condition were then extracted and concatenated.

#### 2.6.2 CTS analysis

To quantify the accuracy of the cortical representation of the attended speech (i.e., CTS), we computed the coherence between source-reconstructed MEG signals and the speech temporal envelope of the target stream (i.e., the narrator’s voice). Speech-brain coherence is a well-established measure of neural tracking (Bourguignon et al., 2013; Peelle et al., 2013; Schmidt et al., 2023; Vander Ghinst et al., 2016) capturing the frequency-specific phase consistency and amplitude association between two signals on a scale from 0 (no linear association) to 1 (full linear association) (Halliday et al., 1995). This was done for all experimental conditions except for the soundless ones (V-only, T-only), where coherence was computed with the temporal envelope of the unheard speech. In T-only, the unheard speech was also the sound used as the basis for the generation of the speech-based vibrotactile stimulation.

The speech temporal envelope was obtained as done for the generation of the vibrotactile signal (*Methods: Stimuli*). The cross-spectral density matrix of the preprocessed MEG data and the speech temporal envelope was computed based on data split into 2-second epochs with a 1.6-second epoch overlap, which afforded a frequency resolution of 0.5 Hz with improved spectral estimation compared with standard 50-% epoch overlap (Bortel & Sovka, 2014). Epochs exceeding 5 pT (magnetometer) or 1 pT (gradiometer) in at least one sensor were classified as artifact-containing and excluded from further analysis. Note that values at a given frequency bin, say *f_b_*, are sensitive to frequencies ~0.5 Hz around, with a sensitivity profile proportional to the Fourier transform of a boxcar function: sinc(π(*f* – *f_b_*)/0.5 Hz).

CTS maps were generated at the source level using each participant’s MRI-based forward model. For that, individual MRI volumetric segmentation was first performed using the FreeSurfer software suite (Fischl, 2012) (Martinos Center for Biomedical Imaging, Boston, MA, RRID:SCR_001847), after which a manual MEG-MRI coregistration was done using the digitized anatomical fiducial landmarks and the head-surface points (MRIlab, MEGIN, Finland). A non-linear transformation of the individual MRIs to the Montreal Neurological Institute (MNI) template brain was then computed using SPM8 (Wellcome Centre for Human Neuroimaging, London, UK, RRID:SCR_007037, http://www.fil.ion.ucl.ac.uk/spm/) and used to project a homogeneous 5-mm grid sampling the MNI brain onto individual MRIs. At each grid point, the currents were modeled as three orthogonally oriented dipoles. The forward model was subsequently computed using the single-layer Boundary Element Method (BEM) approach implemented in MNE-C (Martinos Centre for Biomedical Imaging, Massachusetts, USA) (Gramfort et al., 2014) and reduced to its first two first principal components given the insensitivity of MEG to currents radial to the skull (Bertels et al., 2023). Lastly, the minimum-norm estimation (MNE) inverse solution (Dale & Sereno, 1993) was computed using an MNE regularization parameter based on the consistency condition (Wens et al., 2015). No explicit depth bias correction was done, as coherence values are unaffected by bias correction. Finally, source-level coherence for frequency bins within 0.5-8 Hz was estimated using the dynamic imaging of coherent sources approach (Gross et al., 2001) with the MNE inverse solution. Coherence maps were averaged across frequency bins within the phrasal/sentential (0.5 Hz only), word (1–4 Hz) and syllabic (4–8 Hz) ranges separately. Given that all maps were inherently coregistered to the MNI space, the group-level coherence maps for each condition and CTS frequency were obtained by averaging across participants.

#### 2.6.3 Functional connectivity analysis

We conducted seed-based functional connectivity for the conditions and hemisphere which presented significant CTS values differences (i.e., ATc vs. A-only in the multi-talker background; right STAC) using the phase locking value (PLV), a phase alignment measure ranging from 0 (no synchronization) to 1 (full synchronization) (Aydore et al., 2013). This connectivity measurement was chosen given its robustness to variations in signal amplitude and the demonstrated involvement of phase synchrony in large-scale (i.e., brain regions at >2 cm apart) perceptual brain integration (Aydore et al., 2013; Lachaux et al., 1999; Varela et al., 2001).

In practice, the preprocessed MEG data for each condition were first bandpass filtered into narrow 1 Hz-wide frequency bands. A boxcar window was applied, with cutoff frequencies set to predefined limits ranging from 0.5 to 40 Hz. This filtering process ensured that only the frequency ranges of interest were retained. The filter featured a transition width of 0.01 Hz at the cutoff frequencies, providing precise control over the definition of the passband. MEG noise covariance estimation was performed using a 5-minute recording of empty-room data prior to MNE-based source reconstruction. Next, brain source activity was reconstructed for each condition and within each frequency band with MNE. Seed-based functional connectivity was then measured using PLV with signal orthogonalization for spatial leakage correction (Brookes et al., 2012) from seed to 154 nodes of a dense network-based parcellation of the human brain composed obtained in a former meta-analysis of functional MRI data (Della Penna et al., 2019; Hacker et al., 2013). Each dipole time series was projected onto the direction of its maximum variance (Wens et al., 2014), and the Hilbert transform was applied to derive its analytic signal. The seed was placed at the mean coordinates of the right STAC loci exhibiting significant CTS across the conditions with audible speech. The obtained between-nodes PLV values were averaged within five specific frequency bands (delta: 0.5–4, theta: 4–8, alpha: 8–12, low-beta: 12–21 and high-beta: 21–30 Hz). The latter three were included given their known involvement in speech processing (Biau & Kotz, 2018; Tóth et al., 2019) and because changes in STAC’s synchronization with other brain areas at these non-linguistic frequencies could still facilitate the temporal coordination of neural activity within STAC, optimizing CTS (Hyafil et al., 2015). Of note, each pairwise PLV was computed by using both STAC and the other node as seeds in turn and then averaged (i.e., transpose symmetrisation) (Hipp et al., 2012), to avoid possible asymmetries arising after pairwise orthogonalization.

### 2.7 Statistical analyses

#### 2.7.1 Behavioral performance analysis

Participants’ speech comprehension was analyzed using a logistic mixed-effects regression analysis conducted in R Statistical Software (v4.1.0) (R Core Team, 2021) using the *glmer* function from the *lme4* (Bates et al., 2015) package, with the experimental condition set as the fixed factor and a by-subject random intercept. Binary (yes/no) comprehension responses (correct = 1, incorrect = 0) were used as the dependent variable (four per condition). Video presentation order, within-video condition order (corresponding to content question order), and within-video order assignment (i.e., which predefined condition order was applied to the video) were included as covariates (fixed-effect predictors). A random intercept for video was further added to account for potential stimulus (video-specific) difficulty effects; none of these factors significantly predicted comprehension performance (all *p*’s > .21). Fixed-effects significance was assessed using likelihood ratio tests, with Chi-square distribution test statistics approximation. Since comprehension responses were binary, model estimates were obtained on the log-odds scale using the *emmeans* package. For post hoc comparisons of estimated marginal means, planned one-tailed pairwise contrasts were run in quiet, noise, and matched quiet-noise comparisons using Wald tests. Effect sizes are reported as odds ratios (OR; i.e., the exponentiated log-odds estimates) and corresponding 95% Wald CIs. Multiplicity correction was done using the multivariate t-distribution adjustment.

#### 2.7.2 Assessment of CTS local maxima significance

A non-parametric permutation test (Nichols & Holmes, 2002) was used to assess the statistical significance of the CTS local maxima identified in the group-averaged coherence maps, for each experimental condition and CTS frequency. First, subject-level and group-level *null* CTS maps were generated by computing the coherence between the original MEG signals and a time-shifted version of the attended speech signal in each video. The time-shift involved swapping the first and second halves of each recording, which would practically disrupt any genuine coupling between the attended speech and brain signals, while preserving the spectral properties of the original speech signal. The exact temporal shift applied aligned with a pause in the speech signal, ensuring continuity. Next, group-averaged difference maps were generated by subtracting *genuine* from *null* group-averaged CTS maps; under the null hypothesis, no difference between the two should exist, regardless of the experimental condition. Therefore, the labeling of *genuine* or *null* maps would be interchangeable prior to computing the difference map (Nichols & Holmes, 2002). To reject this null hypothesis and determine the significance level of the observed difference map, the permutation-based sample distribution of the maximum absolute value of the difference maps was obtained from 1000 permutations; the use of this maximum value inherently corrects for multiple comparisons between coherence values. The statistical threshold for significance (*p* < .05) was then set as the 95^th^ percentile of this distribution (Nichols & Holmes, 2002). Finally, all local maxima of CTS exceeding this threshold were interpreted as brain regions exhibiting statistically significant CTS. In conditions where step-down significance testing was used (i.e., T-only for syllabic CTS), significance was assessed iteratively by first identifying the strongest CTS source, then removing its contribution and recomputing the statistical threshold for the remaining sources. As permutation tests may be too conservative for voxels beyond those showing the maximum observed statistic (Nichols & Holmes, 2002) and because CTS can present hemispheric imbalance (Destoky et al., 2019; Vander Ghinst et al., 2016), this procedure was performed separately for each hemisphere. After identifying the coordinates of the local maxima obtained in the group-averaged CTS maps for each experimental condition, individual CTS values were extracted within a 10 mm sphere centred on the group-level coordinates.

#### 2.7.3 Differences in CTS between the experimental conditions

Before evaluating CTS differences between conditions, we ensured that the number of artefact-free epochs used for coherence estimation was comparable across conditions using a one-way repeated-measures ANOVA (*F*(6,174) = 1.38, *p* = .23). We then extracted syllabic, word, and phrasal CTS values from the significant STAC local maxima and analyzed them using linear mixed-effects regression analyses in the R software (R Core Team, 2021), using the *lme4* (Bates et al., 2015) package’s *lmer* function. The experimental condition and hemisphere were set as fixed factors and a by-subject intercept was used. Prior to the analysis, outlier fixing was done by removing CTS values deviating more than 2.5 s.d. from the distribution and setting them to the mean ± 2.5 s.d. Mixed modeling residual normality was checked with residual Q-Q plots and Kolmogorov-Smirnov testing, and model fitting was done using maximum likelihood estimation. Fixed effects were evaluated using F-tests based on Type III sums of squares. Satterthwaite’s method was used to compute the degrees of freedom for the main model’s fixed effects. Two-tailed post-hoc comparisons of the estimated marginal means obtained using the *emmeans* package were performed using paired *t*-tests with multivariate t-distribution multiple comparison correction.

#### 2.7.4 Behavioral relevance of CTS vibrotactile enhancement

To determine whether the improvement in CTS values induced by the vibrotactile input was associated with speech comprehension, we computed a comprehension score as the proportion of correct answers to the four content questions in the ATc experimental condition. For CTS, to isolate the effect of supplemental non-auditory input and mitigate individual baseline auditory variability in unimodal (i.e., A-only) processing, we computed a right-hemispheric benefit score by subtracting the CTS values in the unimodal condition from those in the bimodal condition (CTS_benefit_ = CTS_ATc_ – CTS_A-only_), as in prior investigations of audio-visual CTS enhancement (Haider et al., 2024). We additionally calculated an analogous benefit score for comprehension to further evaluate a benefit—benefit relationship. Given the data count (*n* = 30) and the ordinal nature of the behavioral data (i.e., 0%, 25%, 50%, 75%, or 100%), the associations of interest (i.e., CTS_benefit_ and comprehension_ATc_; CTS_benefit_ and comprehension_benefit_) were assessed using one-tailed (positive) Kendall’s tau rank correlations. All other correlational analyses were performed using one-tailed Pearson’s correlation coefficient.

#### 2.7.5 Multisensory integration

Multisensory integration was evaluated in conditions with significant CTS enhancement (i.e., syllabic CTS in ATc with multi-talker background) using a strict super-additivity criterion (i.e., ATc > A-only + T-only) (Stevenson et al., 2014). To more accurately detect super-additive effects, each participant’s CTS values were first bias-corrected by subtracting the corresponding CTS values obtained in the permutation-derived *null* coherence maps at the same STAC MNI coordinates as in genuine coherence maps (e.g., ATc – ATc_null_). This was done to factor out the positive bias of coherence estimates, which could inflate the A-only + T-only sum and mask super-additive effects. Next, we computed the ATc – (A-only + T-only) difference on the bias-corrected values and tested whether it was greater than 0 using a one-tailed (right-tailed), one-sample *t*-test.

#### 2.7.6 Functional brain connectivity

To investigate between-condition differences in seed-based functional connectivity, group-averaged data were contrasted using mass-univariate paired *t*-tests (one per node-to-node connection, for each frequency band). Significance was assessed with a non-parametric permutation test based on a maximum statistic approach (Nichols & Holmes, 2002). This method tests the null hypothesis of no connectivity difference between two conditions by permuting subject-level condition labels and creating a difference map. Here, 2000 permutations were performed to generate the distribution of the contrast coefficients of the maximum absolute values across all the connections in the difference map. The significance threshold was then established as the 95^th^ percentile of the obtained null distribution (α = .05), a procedure that inherently corrects for multiple comparisons. Observed *t*-statistics exceeding this threshold were interpreted as significant functional connectivity differences between experimental conditions.

## 3 Results

### 3.1 Behavioral comprehension evaluation

**Fig. 3.**
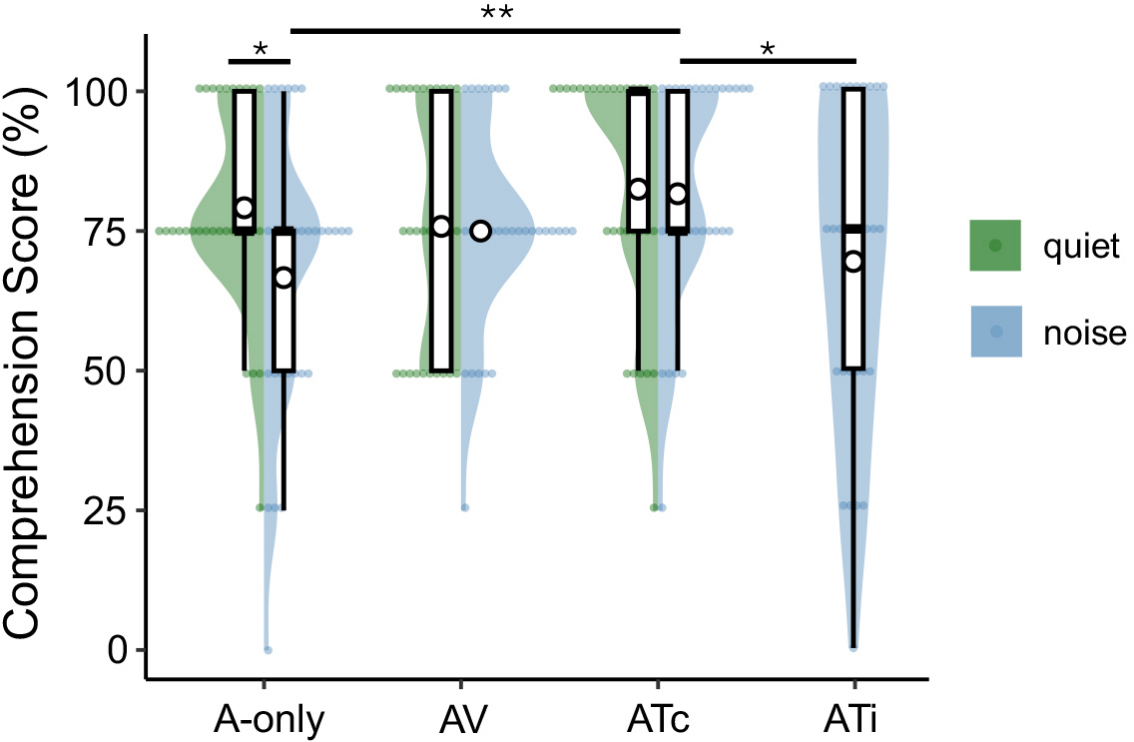
*Comprehension scores (% correct) across experimental conditions.* Boxplots represent interquartile range (IQR), median (horizontal line), mean (white circle), and outliers (points outside of whiskers). Conditions with no visible boxplots reflect an IQR of 0. Individual data points represent the percentage of correct answers to the four yes/no content questions for each experimental condition. A-only: auditory-only, AV: audio-visual, ATc: audio-tactile with congruent vibrations, ATi: audio-tactile with incongruent vibrations. Significance marked for pairwise one-tailed testing: *p < .05, **p < .01.

After watching each of the four videos (Fig. 1), participants answered sets of forced-choice yes/no content questions to assess comprehension of the speech material in each experimental condition (i.e., A-only, AV, ATc in quiet; A-only, AV, ATc, ATi in the multi-talker background; T-only and V-only). Figure 3 displays the distribution of mean comprehension scores in conditions with heard speech, computed as the number of correct answers per condition across all videos. Mean accuracy ranged from 66.7% to 82.5%, suggesting consistent task engagement. Although comprehension in the A-only quiet condition was not at ceiling (79.2 ± 18.4%; mean ± s.d.), performance in all conditions was significantly above the 50% chance level imposed by the yes/no comprehension questions format (all *t*(30) > 3.73, *p*’s < .001). The absence of a ceiling effect in quiet likely reflects the complexity of the continuous speech material, which featured information-rich content, and the extensive length of the videos (*Methods: Stimuli*). In contrast, mean performance in soundless conditions (T-only, V-only; 44.2% for both) revealed that, in the absence of auditory speech information, participants relied on guessing; these conditions were hence excluded from the analysis of comprehension accuracy.

A logistic mixed-effects regression analysis of the participants’ answers to the content questions revealed that comprehension varied significantly across the experimental conditions (χ²(6) = 15.75, *p* = .015). Following this, we conducted post-hoc pairwise comparisons across conditions. For each contrast, odds ratios (OR) > 1 indicate higher odds of correct responses in the first mentioned condition. As expected, the multi-talker background significantly impaired comprehension in the unimodal condition (quiet vs. noise in A-only: 12.5% decrease, odds ratio (OR) = 2.13, 95% CI [1.01, 4.47], *z* = 2.43, *p*_adj_ = .023). This deleterious effect of noise was absent when a supplemental congruent non-auditory sensory input was provided (quiet vs. noise in AV: OR = 0.93, 95% CI [0.43, 2.01], *z* = 0.23, *p*_adj_ = .94; quiet vs. noise in ATc: OR = 1.11, 95% CI [0.49, 2.52], *z* = 0.29, *p*_adj_ = .77).

Across the quiet conditions, neither seeing the face of the attended speaker nor sensing a congruent vibration impacted comprehension (AV vs. A-only: OR = 0.73, 95% CI [0.33, 1.57], *z* = -0.98, *p*_adj_ = .99; ATc vs. A-only: OR = 1.3, 95% CI [0.59, 2.87], *z* = 0.79, *p*_adj_ = .46). In the multi-talker background, visual speech provided a modest but not statistically significant increase in comprehension (AV vs. A-only: 8.3% improvement, OR = 1.66, 95% CI [0.78, 3.53], *z* = 1.68, *p*_adj_ = .18), while the vibrotactile stimulation significantly improved performance only when congruent with the attended speech (ATc vs. A-only: 15% improvement, OR = 2.5, 95% CI [1.12, 5.58], *z* = 2.88, *p*_adj_ = .009; ATi vs. A-only: 3.3% improvement, OR = 1.18, 95% CI [0.58, 2.4], *z* = 0.59, *p*_adj_ = .73). The difference in performance between the two AT conditions was also significant (ATc vs. ATi: OR = 2.11, 95% CI [0.94, 4.73], *z* = 2.33, *p*_adj_ = .044). However, the difference between ATc and AV performance was not significant, neither in quiet (ATc vs. AV: 6.7% difference, OR = 0.55, 95% CI [0.24, 1.26], *z* = 1.67, *p*_adj_ = .99), nor in noise (ATc vs. AV: 6.7% difference, OR = 0.66, 95% CI [0.29, 1.49], *z* = 1.28, *p*_adj_ = .99).

Overall, speech comprehension in quiet was unaffected by the addition of a supplemental, non-auditory congruent sensory signal, whether visual or tactile. Most importantly, congruent speech-based vibrations (ATc) mitigated the detrimental effect of the multi-talker background observed in the unimodal (A-only) condition, whereas this effect was not significant in the AV condition.

### 3.2 Cortical tracking of speech (CTS)

**Fig. 4.**
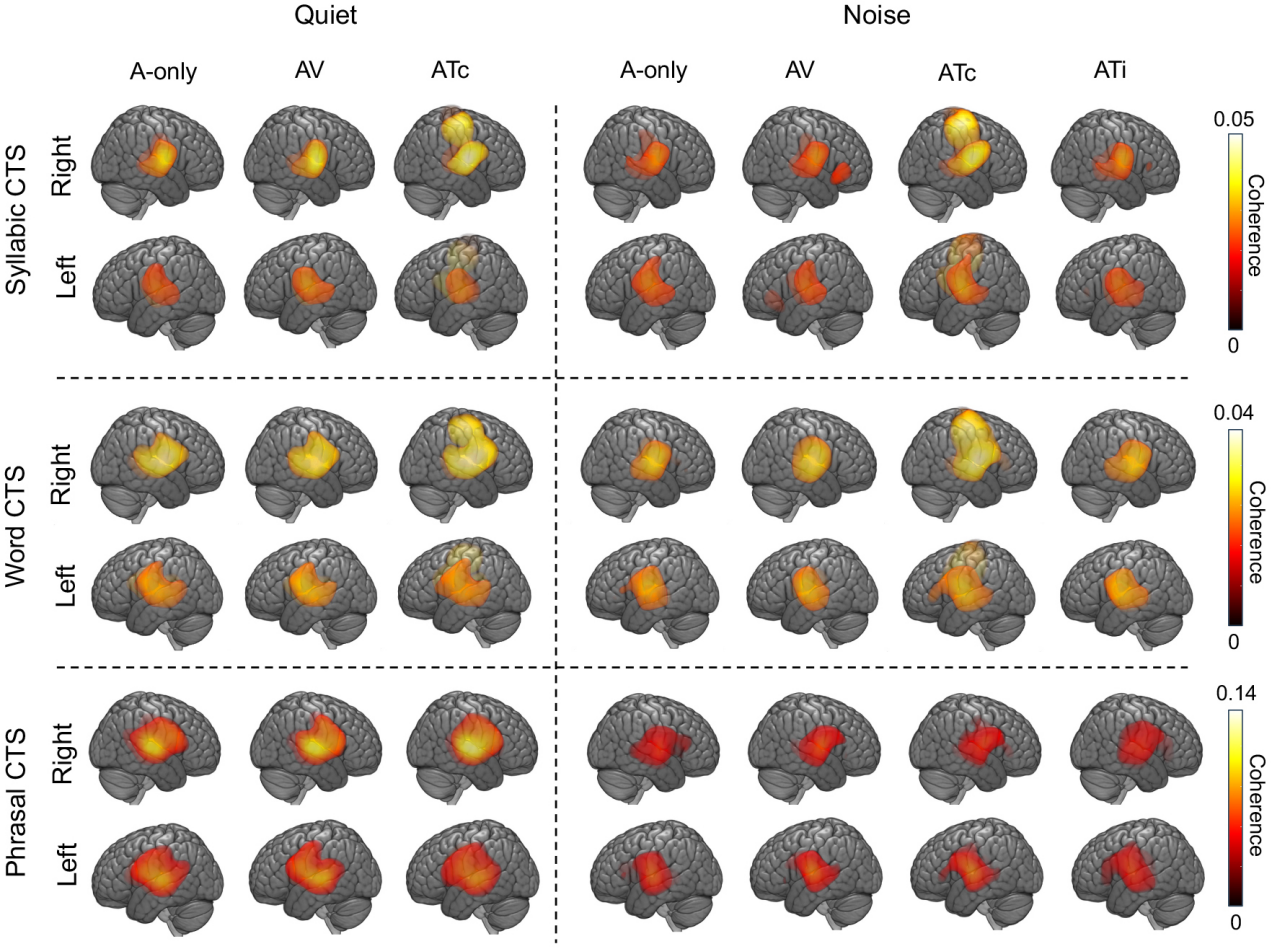
Brain distribution of significant cortical tracking of speech (CTS) at syllabic (top), word (middle), and phrasal (bottom) levels, in quiet (left) and with multi-talker background noise (right). Group-level coherence maps (n = 30) obtained by statistically masking above the significance threshold level with the use of nonparametric permutation statistics. A-only: auditory-only, AV: audio-visual, ATc: audio-tactile with congruent vibrations, ATi: audio-tactile with incongruent vibrations.

**Fig. 5.**
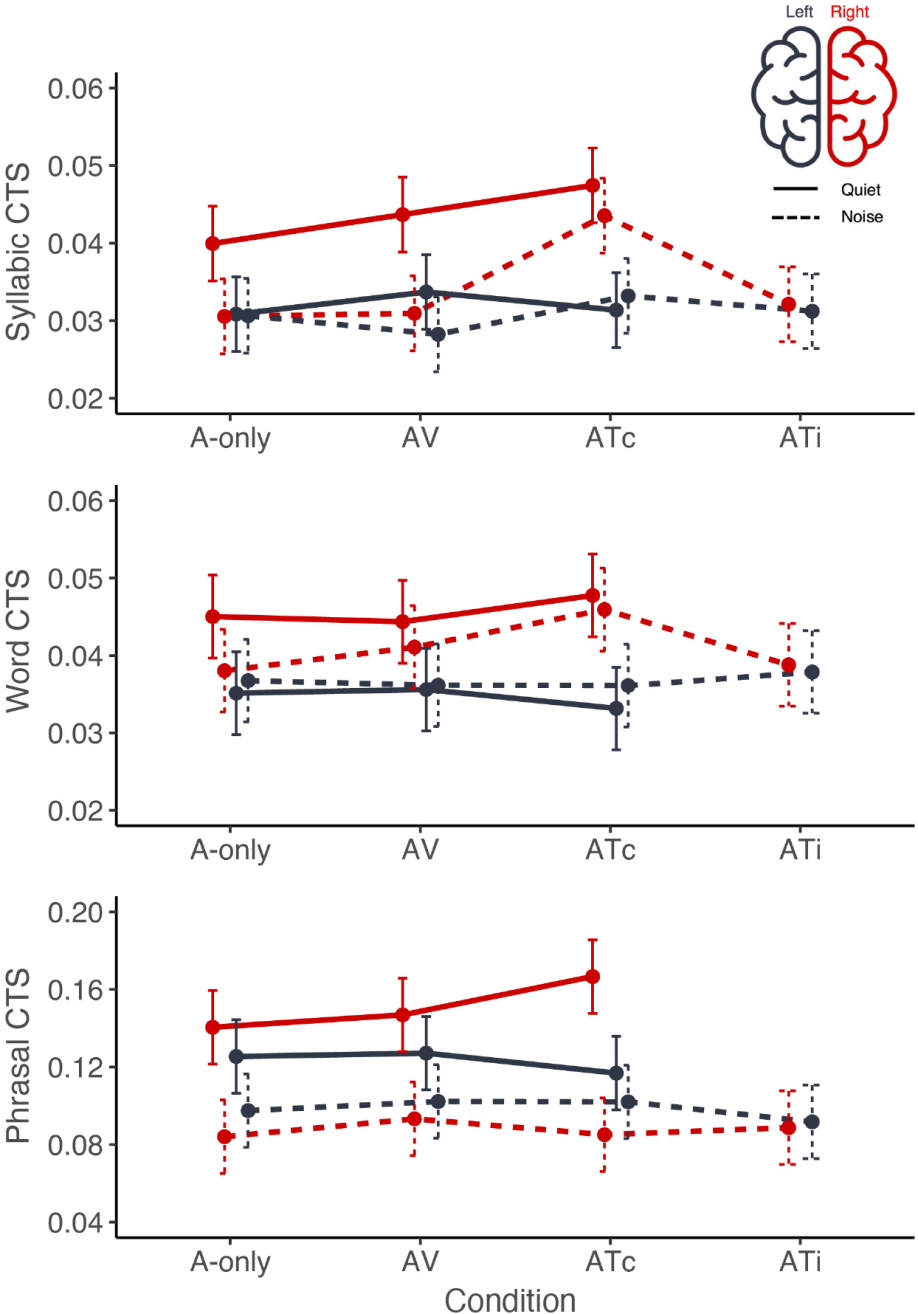
Impact of experimental condition and hemisphere on the cortical tracking of speech (CTS) at the syllabic (top), word (middle), and phrasal (bottom) rates. Means are depicted by the circles and 95% confidence intervals by the vertical lines. Line patterns correspond to the auditory conditions (connected traces – quiet, dashed traces – noise), color indicates the hemisphere in which the CTS was measured (left hemisphere – blue, right hemisphere – red). A-only: auditory-only, AV: audio-visual, ATc: audio-tactile with congruent vibrations, ATi: audio-tactile with incongruent vibrations.

Next, we examined how CTS varied across conditions by computing the coherence between the attended speech temporal envelope and source-localized brain signals, at syllabic (i.e., 4–8 Hz), word (1–4 Hz), and phrasal/sentential (<1 Hz) rates. Figure 4 illustrates the cortical distribution of CTS across all experimental conditions, for all three frequency rates. In all cases, CTS sources were present bilaterally in supratemporal auditory cortices (STAC) (see Table 1 for MNI coordinates and statistical significance of the STAC local maxima), confirming the central role of auditory regions in CTS and speech processing. Given our assumption that improvements in speech comprehension in AT conditions stem from CTS refinement in auditory regions, we next analyzed CTS values obtained across conditions at bilateral STAC (Fig. 5) using linear mixed-effects modeling, separately for each frequency. *Post-hoc* pairwise statistical comparisons (two-tailed) following these analyses can be found in Table 2.

**Table 1.** Significant local maxima of cortical tracking of speech (CTS): peak MNI coordinates [mm], peak coherence values, significance level, and anatomical location.

		Syllabic CTS			Word CTS			Phrasal CTS		
Condition		Coord. [mm]	Coh.	Region	Coord. [mm]	Coh.	Region	Coord. [mm]	Coh.	Region
A-only	Quiet	[45 –10 10]	0.036^***^	R STAC	[45, -11, 11]	0.039^***^	R STAC	[49 –20 8]	0.12^***^	R STAC
		[–38 –16 11]	0.027^***^	L STAC	[-41, -14, 11]	0.031^***^	L STAC	[–44 –21 6]	0.10^***^	L STAC
	Noise	[49 –8 13]	0.028^***^	R STAC	[48, -9, 12]	0.034^***^	R STAC	[–41 –12 7]	0.08^***^	L STAC
		[–43 –14 16]	0.027^***^	L STAC	[-43, -13, 12]	0.032^***^	L STAC	[50 –11 4]	0.06^***^	R STAC
		[53 –43 10]	0.02^*^	R TPJ	[50, 33, 3]	0.021^*^	R IFG	[47 28 17]	0.04^*^	R IFG
					[-42, 25, 8]	0.021^*^	L IFG			
AV	Quiet	[43 –9 10]	0.04^***^	R STAC	[47, -14, 7]	0.039^***^	R STAC	[48 –19 6]	0.13^***^	R STAC
		[–41 –14 12]	0.029^***^	L STAC	[-41, -13, 9]	0.031^***^	L STAC	[–46 –23 4]	0.10^***^	L STAC
	Noise	[47 –7 14]	0.027^***^	R STAC	[42, -12, 11]	0.037^***^	R STAC	[–44 –13 8]	0.086^***^	L STAC
		[–41 –16 12]	0.025^***^	L STAC	[-43, -11, 10]	0.032^***^	L STAC	[43 –11 7]	0.076^***^	R STAC
		[49 33 –4]	0.021^**^	R IFG				[–32 25 7]	0.045^*^	L FO
ATc	Quiet	[49 –6 11]	0.043^***^	R STAC	[48, -13, 9]	0.042^***^	R STAC	[45 –17 6]	0.14^***^	R STAC
		[38 –17 52]	0.039^***^	R S1	[38, -18, 50]	0.039^***^	R S1	[–43 –23 4]	0.096^**^	L STAC
		[–40 –17 10]	0.028^***^	L STAC	[-41, -17, 5]	0.029^***^	L STAC	[–39 –5 20]	0.083^**^	L I
					[-38, -6, 19]	0.029^***^	L I			
	Noise	[39 –18 54]	0.042^***^	R S1	[49, -7, 12]	0.04^***^	R STAC	[–49 –11 8]	0.08^***^	L STAC
		[52 –3 15]	0.039^***^	R STAC	[35, -18, 50]	0.04^***^	R S1	[53 –5 15]	0.076^***^	R STAC
		[–43 –13 14]	0.03^***^	L STAC	[-39, -13, 6]	0.031^***^	L STAC	[–41 28 5]	0.051^*^	L IFG
					[-44, 31, 4]	0.023^*^	L IFG			
ATi	Noise	[48 –7 15]	0.029^***^	R STAC	[43, -11, 10]	0.034^***^	R STAC	[–39 –7 15]	0.07^***^	L STAC
		[–38 –15 16]	0.027^***^	L STAC	[-41, -10, 13]	0.033^***^	L STAC	[47 –16 7]	0.07^***^	R STAC
		[53 30 3]	0.02^*^	R IFG						
V-only								[43, -35, 17]	0.035^*^	R PSTG
T-only		[43, -17, 59]	0.041^***^	R S1	[40, -17, 58]	0.041^***^	R S1	[63, -13, 11]	0.037^*^	R STG
		[14, -20, 76]	0.032^*^	R PMC	[51, -19, 14]	0.029^*^	R STG			
		[59, -4, 17]	0.027^†^	R STG	[-24, -7, 16]	0.024^*^	L I			
					[-52, -28, 27]	0.024^*^	L SMG			

Significance level: *p < .05, **p < .01, ***p < .001; † denotes significance level obtained using a stepwise procedure (nonparametric permutation statistics). A-only: auditory-only, AV: audio-visual, ATc: audio-tactile with congruent vibrations, ATi: audio-tactile with incongruent vibrations, R: right, L: left, STAC: supratemporal auditory cortex, FO: frontal operculum, IFG: inferior frontal gyrus, I: insular cortex, S1: primary somatosensory cortex, SMG: supramarginal gyrus, TPJ: temporoparietal junction, PMC: premotor cortex, Coh.: coherence, Coord.: coordinates.

**Table 2.** Pairwise comparisons of syllabic, word, and phrasal cortical speech tracking (CTS) values at auditory cortices.

		Contrast	Syllabic CTS			Word CTS			Phrasal CTS		
Env.	Hemisphere		*t*_403_	*p*_unc_	*p*_adj_	*t*_403_	*p*_unc_	*p*_adj_	*t*_403_	*p*_unc_	*p*_adj_
Quiet	Left	A-only vs. ATc	-0.210	0.83	1	0.644	0.52	1	0.730	0.46	1
		A-only vs. AV	-1.161	0.25	0.99	-0.152	0.88	1	-0.146	0.88	1
		AV vs. ATc	0.951	0.34	1	0.796	0.43	1	0.876	0.38	1
	Right	A-only vs. ATc	-3.048	0.003	0.06	-0.887	0.38	1	-2.227	0.027	0.42
		A-only vs. AV	-1.518	0.13	0.90	0.216	0.83	1	-0.543	0.59	1
		AV vs. ATc	-1.530	0.13	0.90	-1.104	0.27	0.99	-1.684	0.093	0.82
Noise	Left	A-only vs. ATc	-1.041	0.30	0.99	0.210	0.83	1	-0.386	0.69	1
		A-only vs. ATi	-0.235	0.81	1	-0.365	0.72	1	0.493	0.62	1
		A-only vs. AV	0.973	0.33	0.99	0.193	0.85	1	-0.402	0.69	1
		ATc vs. ATi	0.806	0.42	0.99	-0.575	0.57	1	0.879	0.38	1
		AV vs. ATc	-2.014	0.045	0.58	0.017	0.99	1	0.016	0.987	1
	Right	A-only vs. ATc	-5.282	<.0001	**<.0001**	-2.574	0.01	0.2	-0.090	0.928	1
		A-only vs. ATi	-0.642	0.52	1	-0.247	0.81	1	-0.399	0.690	1
		A-only vs. AV	-0.161	0.87	1	0.992	0.32	1	-0.785	0.433	1
		ATc vs. ATi	4.640	<.0001	**0.0001**	2.327	0.02	0.35	-0.308	0.758	1
		AV vs. ATc	-5.120	<.0001	**<.0001**	-1.582	0.11	0.87	0.695	0.488	1
Cross-environment	Left	Quiet vs. Noise (A-only)	0.084	0.93	1	-0.531	0.60	1	2.378	0.018	0.31
		Quiet vs. Noise (ATc)	-0.747	0.46	1	-0.965	0.33	1	1.262	0.208	0.97
		Quiet vs. Noise (AV)	2.218	0.027	0.42	-0.187	0.85	1	2.122	0.034	0.49
	Right	Quiet vs. Noise (A-only)	3.820	0.0002	**0.004**	2.281	0.02	0.38	4.806	<.0001	**0.0001**
		Quiet vs. Noise (ATc)	1.586	0.11	0.87	0.594	0.55	1	4.564	<.0001	**<.0001**
		Quiet vs. Noise (AV)	5.176	<.0001	**<.0001**	1.072	0.28	0.99	6.943	<.0001	**0.0002**

*P*-values reflect two-tailed pairwise comparisons of estimated marginal means derived from linear mixed-effects modeling, uncorrected (unc) and adjusted (adj) for multiplicity with the multivariate t-distribution approach. Values in bold represent statistically significant pairwise comparisons after adjustment for multiplicity. A-only: auditory-only, AV: audio-visual, ATc: audio-tactile with congruent vibrations, ATi: audio-tactile with incongruent vibrations, Env: auditory environment.

For syllabic CTS, we uncovered a significant main effect of the experimental condition (*F*(6,390) = 11.85, *p* < .0001), of the measured hemisphere (*F*(1,390) = 58.73, *p* < .0001), and a significant interaction between the two (*F*(6,390) = 6.06, *p* < .0001). The main effect of hemisphere indicated that syllabic CTS was overall right-lateralized (left vs. right; *t*(403) = -7.54, *p*_adj_ < .0001). The hemispheric-specific impact of the multi-talker background further revealed a significant syllabic CTS decrease in the right hemisphere in A-only (*p*_adj_ = .004) and AV (*p*_adj_ < .0001), but, critically, not in ATc (*p*_adj_ = .87). In the left hemisphere, the multi-talker background did not impact syllabic CTS in either condition (all *ps*_adj_ > .42). Additionally, in quiet, no significant difference between A-only, AV, and ATc conditions emerged in either hemisphere (all *ps*_adj_ > .06), although a tendency for higher right-hemispheric syllabic CTS in ATc compared to A-only could be observed (*p*_unc_ = .003). In the multi-talker background, the same pattern emerged in the left hemisphere (i.e., no difference between A-only, AV, ATc, and ATi; all *ps*_adj_ > .58), but, distinctively, not in the right hemisphere, where CTS was higher in ATc compared to A-only (*p*_adj_ < .0001), AV (*p*_adj_ < .0001), and ATi (*p*_adj_ < .0001). These results suggest that, while the presence of a multi-talker background can significantly degrade right-hemispheric syllabic-level CTS, congruent vibrotactile input (ATc) effectively mitigates this degradation.

For word-level CTS, we observed a significant main effect of hemisphere (*F*(1,390) = 39.67, *p* < .0001) and an interaction between condition and hemisphere (*F*(6,390) = 2.77, *p* = .012), with no significant main effect of the experimental condition (*F*(6,390) = 0.76, *p* = .61). Overall, word CTS was right-lateralized (left vs. right; *t*(403) = -6.19, *p*_adj_ < .0001). This lateralization varied depending on the experimental condition, driving the significant interaction between condition and hemisphere. In quiet, it was present in A-only (*t*(403) = -3.23, *p*_adj_ = .033) and ATc conditions (*t*(403) = -4.76, *p*_adj_ = .0001), but not in AV (*t*(403) = -2.86, *p*_adj_ = .098). In the multi-talker background, lateralization was maintained only in ATc conditions (*t*(403) = -3.20, *p*_adj_ = .036; *ps*_adj_ for remaining conditions > .86). In line with previous research (Vander Ghinst et al., 2019), word-level CTS was robust to the presence of the multi-talker background, with no significant quiet—noise CTS decrease in either modality (A-only, AV and ATc) or hemisphere (all *ps*_adj_ > .38). Additionally, a trend-level right-hemispheric enhancement of CTS was present with congruent vibrations in multi-talker conditions (*p*_unc_ = .01); this effect did not survive multiplicity correction. Therefore, within hemisphere, word CTS did not differ between modalities in either quiet (all *ps*_adj_ > .99) or multi-talker conditions (all *ps*_adj_ > .2). These findings indicate that, apart from an impact on CTS lateralization, word CTS is not significantly degraded by the presence of a multi-talker background and demonstrates no robust within-hemisphere modality differences.

For phrasal CTS, we observed a significant main effect of the experimental condition (*F*(6,390) = 17.1, *p* < .0001), as well as a significant interaction between condition and hemisphere (*F*(6,390) = 4.25, *p* = .00037), but no significant effect of the hemisphere (*F*(1,390) = 1.9, *p* = .17). In the right hemisphere, the multi-talker background markedly reduced phrasal CTS compared to quiet in A-only conditions (*p*_adj_ = .0001). This effect persisted even with visual speech (AV; *p*_adj_ = .0002) and congruent tactile vibrations (ATc; *p*_adj_ < .0001). In the left hemisphere, the noise dampening effect did not reach multiple comparison correction significance in any condition. Uncorrected effects were, however, observed in A-only (*p*_unc_ = .018) and AV (*p*_unc_ = .034) conditions, but not in the ATc condition (*p*_unc_ = .21), suggesting that the vibrotactile input may have slightly attenuated the impact of noise at this timescale and hemisphere. Separately for quiet and the multi-talker background conditions, CTS did not differ significantly between A-only, AV, ATc, and ATi in either hemisphere (all *ps*_adj_ > .42). Overall, we found a robust right-hemispheric reduction in phrasal CTS due to the multi-talker background, which was not significantly countered by either visual speech or speech-based vibrations.

Cumulatively, these findings confirm the detrimental effect of competing background talkers on both phrasal and syllabic CTS of the attended speaker at the STAC. Chiefly, they underscore the key enhancement of syllabic CTS at the right STAC induced by congruent speech-derived vibrotactile stimulation in the multi-talker background.

### 3.3 Linking audio-tactile CTS enhancement to speech comprehension

**Fig. 6.**
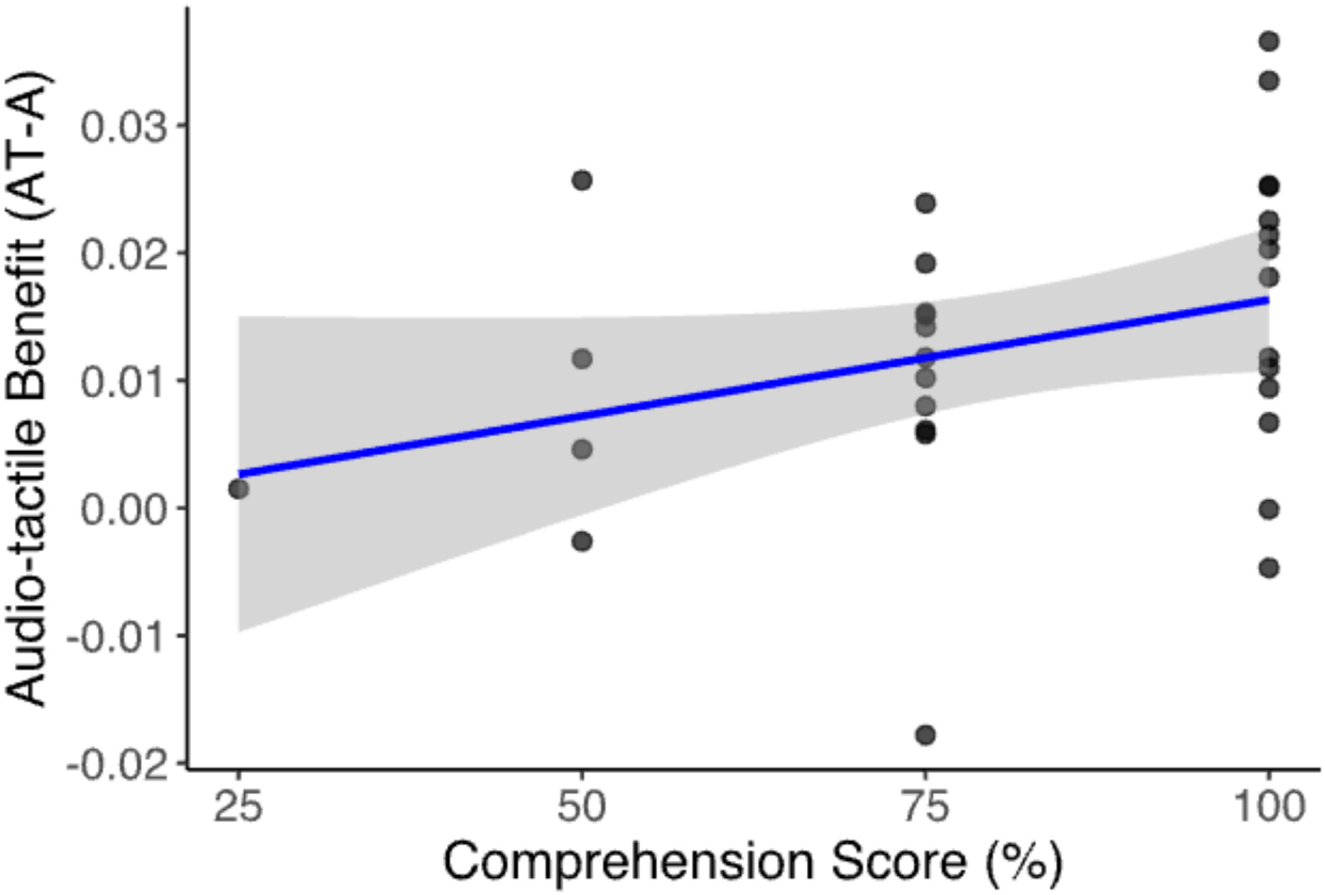
Behavioural relevance of audio-tactile (AT) cortical tracking of speech (CTS) enhancement. Correlation between the syllabic CTS increase in right-hemispheric supratemporal auditory cortex in the ATc condition (relative to the auditory-only condition) and comprehension score in the same condition.

After uncovering significant audio-tactile enhancing effects in both behavioral and CTS measures, we investigated whether these effects were linked. First, we assessed whether the ATc CTS increase in the multi-talker condition was behaviorally relevant. To do so, we tested for a positive correlation between the right STAC syllabic CTS increase in the ATc (i.e., ATc – A-only) and speech comprehension performance in ATc (percentage of correct responses to the four condition-specific comprehension questions). This analysis revealed a significant positive correlation (Fig. 6; τ(28) = 0.27, *p* = .03). Next, to evaluate if the CTS benefit provided by the vibrotactile stimulation in ATc correlated with the comprehension benefit (i.e., the ATc–A-only difference in comprehension performance), we tested the association of the two measures and found no significant link (τ(28) = -0.07, *p* = .68). Thus, despite the absence of a direct benefit-benefit correlation, the syllabic CTS enhancement at right STAC provided by congruent speech vibrations was linked to increased speech understanding, consistent with a brain-behavior association.

### 3.4 Extra-auditory CTS sources

Several loci exhibiting significant CTS outside STAC, previously implicated in CTS (Bertels et al., 2023; Vander Ghinst et al., 2016, 2021), were observed, particularly in multi-talker conditions (Table 1), where weaker CTS sources can attain significance during permutation testing (*Methods: Statistical Analyses*). More importantly, however, in ATc conditions (both in quiet and the multi-talker background), we found significant syllabic and word CTS sources in the right primary somatosensory (S1) cortex (Fig. 4), which likely reflected the direct somatosensory processing of the left-hand vibrotactile stimulation. Given this, we next examined if individual differences in this cortical somatosensory processing were related to the observed syllabic AT CTS benefit at STAC. More specifically, we sought to find whether the latter was driven by the processing of the vibrotactile input in the right S1 cortex—potentially reflecting better intrinsic sensory encoding—rather than being STAC-specific. Correlational analyses indicated no association between syllabic CTS at the S1 cortex and either the ATc syllabic CTS benefit (*r*(28) = 0.09, *p* = .31) or the ATc syllabic CTS values (*r*(28) = 0.17, *p* = .19). As such, these findings suggest that the observed CTS benefit was not driven by a somatosensory encoding-related mechanism but rather resulted from an auditory-specific CTS enhancement.

### 3.5 CTS in soundless conditions

**Fig. 7.**
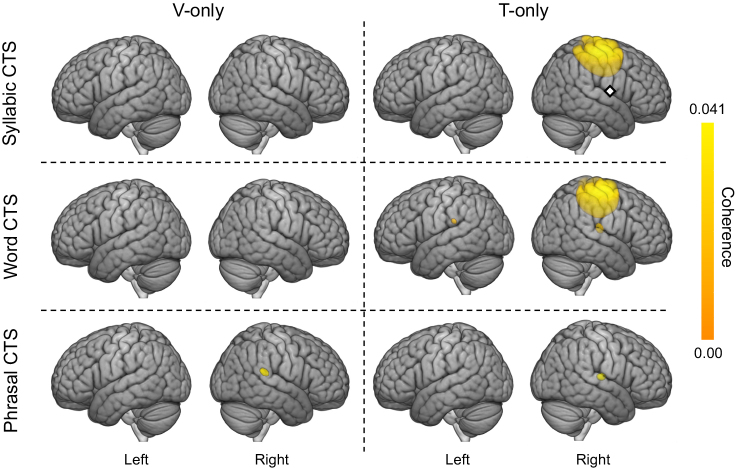
Brain distribution of significant cortical tracking of speech (CTS) in the soundless conditions: video-only (V-only, left) and tactile-only (T-only, right) for the syllabic (top), word (middle) and phrasal (bottom) rates. Group-level coherence maps (n = 30) obtained by statistically masking above the significance threshold level with the use of nonparametric permutation statistics. CTS was computed between brain signals and the unheard speech envelope. Rhombus symbol denotes a CTS source obtained using a step-down procedure. For visualization, only sources with |MNI x| ≥ 25 are shown.

To better uncover how unimodal speech-derived non-auditory inputs influence CTS, we computed CTS in the soundless conditions (V-only and T-only) using the speech envelope of the unheard speech signal at syllabic, word, and phrasal frequencies. In T-only, this envelope corresponded to the envelope of the vibrotactile stimulation. Significant phrasal CTS was observed in both conditions (all MNI coordinates found in Table 1), exclusively in the right hemisphere but at partially distinct locations near STAC, that is, in the posterior superior temporal gyrus (STG) in V-only and in the middle portion of the STG in T-only (Fig. 7). Conversely, significant syllabic and word CTS sources were observed exclusively in T-only. For syllabic CTS, these were localized at the right S1 and premotor cortices, while for word CTS, these were present at the right S1 and STG, along with the left insular and inferior parietal/supramarginal areas.

For the syllabic CTS T-only condition, we ran an additional step-down procedure for the cluster significance computation, whereby the largest significant cluster (obtained based on the *p*-value) was iteratively removed from analysis and the remaining clusters were tested against an adjusted significance threshold (Nichols & Holmes, 2002). This approach was motivated by the right-hemispheric STG cluster uncovered for word CTS in T-only and the assumption that somatosensory-specific syllabic CTS likely overshadowed the more subtle CTS in auditory regions during the maximum statistics significance computation. This step-down analysis, indeed, revealed an additional right STG syllabic CTS local maximum situated close to STAC. This T-only syllabic CTS at right STAC, while significantly lower than A-only CTS in quiet (*t*(29) = -2.45, *p* = .02), did not differ significantly from CTS in the multi-talker background (*t*(29) = 0.15, *p* = .89). It was, however, lower than CTS in both ATc conditions (quiet: *t*(29) = -3.4, *p* = .002; multi-talker: *t*(29) = -4.06, *p* < .001), suggesting that syllabic CTS at STAC is enhanced in ATc conditions beyond T-only CTS.

### 3.6 Multisensory integration

At syllabic rates, CTS in multi-talker ATc exceeded both A-only and T-only CTS at STAC, which was suggestive of multisensory integration using a *max criterion* (ATc larger than either unimodal condition) (Stevenson et al., 2014). Since for population measures (e.g., M/EEG) this can arise from areal convergence (i.e., distinct responses in unisensory neural populations recorded together) (Venezia et al., 2015), we implemented a more stringent *super-additive* multisensory integration criterion. More precisely, we investigated whether CTS in the multisensory condition (here, ATc) surpassed the sum of activity observed during the corresponding unimodal conditions (A-only and T-only; ATc > A-only + T-only) (Holmes & Spence, 2005; Stevenson et al., 2014). In practice, we subtracted the sum of the bias-corrected (see *Methods*) CTS in the two unisensory conditions from the one in ATc (i.e., ATc – (A-only + T-only)). Results revealed that this difference was not significantly larger than 0 (one-tailed *t*(29) = 0.37, *p* = .36), providing no conclusive evidence for the super-additivity of our effects.

### 3.7 Functional connectivity changes linked to audio-tactile CTS enhancement

Lastly, we examined if the significant syllabic CTS enhancement in the multi-talker ATc condition (vs. A-only) was accompanied by broader functional connectivity changes of the right STAC. We hypothesized that, in the ATc condition, this area synchronized more strongly with associative cortical areas involved in AT multisensory integration (Soto-Faraco & Deco, 2009), or with somatosensory cortices (as formerly shown with visual cortices in AV speech (Peelle et al., 2022)). To address this, we conducted a seed-based functional connectivity analysis at the right STAC local maxima, comparing multi-talker ATc and A-only conditions.

**Fig. 8.**
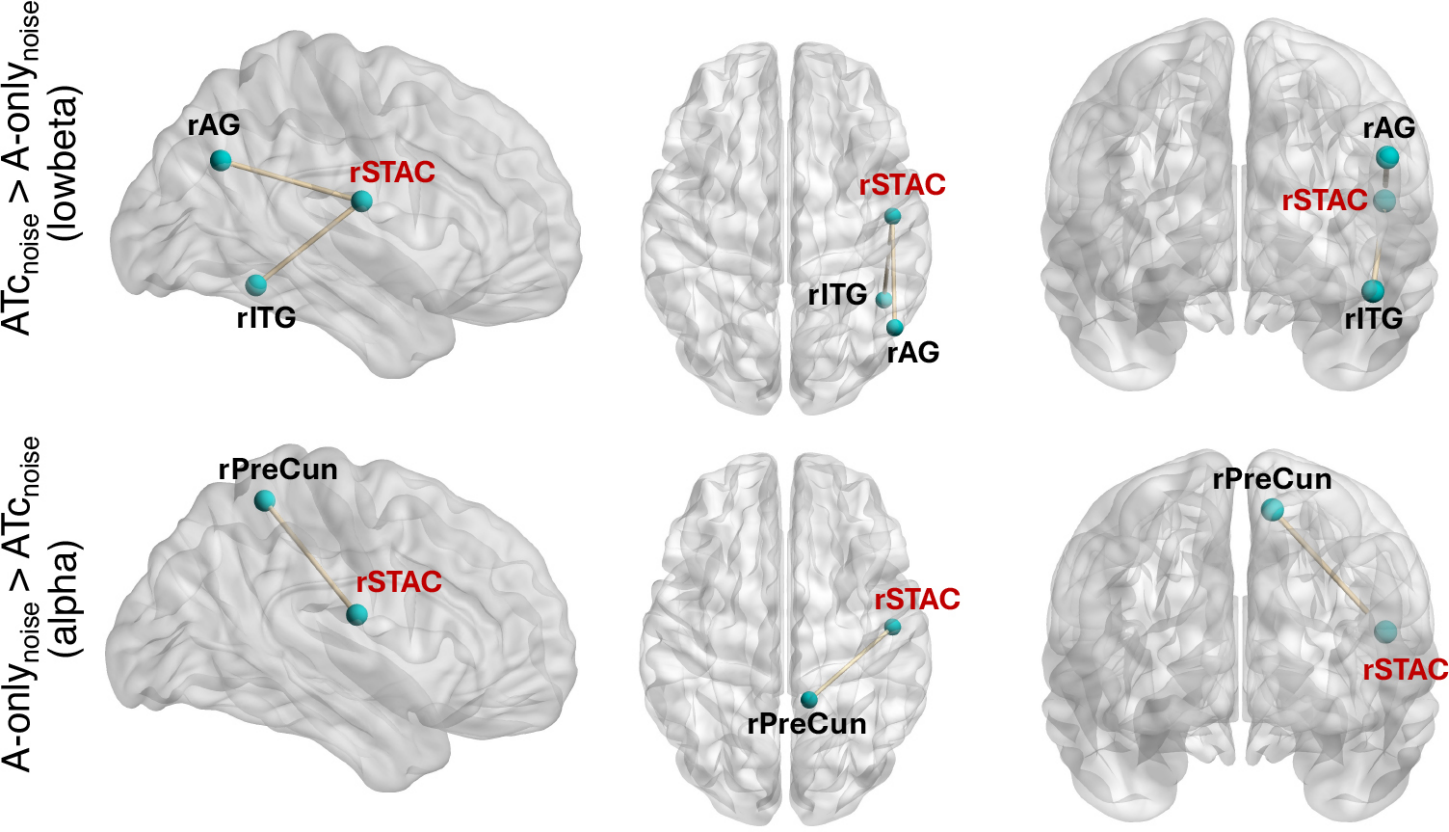
Significant functional connectivity changes in the audio-tactile multi-talker condition compared to the unimodal, auditory-only condition. Functional connectivity changes seeded from CTS local maximum in the right supratemporal auditory cortex (STAC, in red). r = right hemisphere, AG = angular gyrus, ITG = inferior temporal gyrus, PreCun = precuneus, A-only = auditory-only, ATc = audio-tactile with congruent vibrations. Figures created using BrainNet viewer (Xia et al., 2013).

Results (Fig. 8) revealed that the addition of the congruent speech-based vibrotactile stimulation was not accompanied by right STAC functional connectivity changes with somatosensory-specific regions in any frequency band. It was, however, associated with an increased low beta-band functional connectivity with the right angular gyrus (AG: [51, –64, 32] mm; *p* ≲ .05, 2000 permutations), as well as with the posterior part of the right inferior temporal gyrus (ITG: [46, –51, –14] mm; *p* ≲ .05, 2000 permutations). Additionally, we observed a significant decrease in alpha-band connectivity with the precuneus ([11, –46, 59] mm; *p* ≲ .05, 2000 permutations). A secondary analysis seeded from the left STAC comparing the same conditions (i.e., multi-talker ATc with A-only) did not uncover any significant functional connectivity differences between the two in any frequency band.

Overall, these findings indicate that in the ATc compared with the A-only condition, right STAC functional connectivity is increased specifically with higher-order, ipsilateral, associative parietal, and temporal neocortical regions, rather than with somatosensory-specific areas, potentially reflecting enhanced supramodal integration mechanisms.

## 4 Discussion

This study demonstrates that, in a complex multi-talker auditory scene, the concurrent presentation of congruent speech-derived vibrotactile stimulation is associated with increased CTS of attended speech syllabic rhythms in the right STAC. Behaviorally, comprehension in this audio-tactile condition improved relative to A-only and positively correlated with the AT syllabic CTS increase. This effect was accompanied by frequency-specific changes in functional connectivity between the right STAC and ipsilateral, higher-order associative parieto-temporal neocortical areas, suggesting the complementary emergence of a broader, long-range network-level modulation during audio-tactile speech processing.

### 4.1 Vibrotactile enhancement of speech comprehension

Behaviorally, comprehension of the attended speaker in the multi-talker background improved significantly (15%) when listeners received speech-derived vibrotactile stimulation congruent with the attended speech stream. These results align with previous reports of behavioral, sentence-level vibrotactile comprehension enhancement (Guilleminot & Reichenbach, 2022; Răutu et al., 2023), further highlighting that this multisensory effect can emerge even during naturalistic, connected speech listening. In quiet, the impact of the vibrations was markedly reduced (3.3%) and non-significant, indicating its contingency on the presence of background noise. The noise-specificity of this effect conforms to the inverse effectiveness principle of multisensory integration, which states that as response accuracy to unimodal stimuli decreases, the strength of multisensory integration increases (Meredith & Stein, 1986). Incongruent vibrations also did not yield a significant benefit compared to A-only, underscoring the added essential role of synchronicity between sensory modalities (i.e., temporal rule of multisensory integration) (Holmes & Spence, 2005). This corroborates previous observations showing significant tactile speech enhancement with congruent, but not incongruent, vibrotactile speech input (Schulte et al., 2023). Importantly, the emergence of this benefit in untrained listeners with no prior exposure to the vibrotactile stimulation reinforces theories that conceptualize speech perception as a multimodal sensory phenomenon, with modality-invariant speech cues aiding comprehension regardless of the transmission channel (Fowler & Dekle, 1991; Rosenblum, 2008; von Kriegstein, 2012).

### 4.2 Vibrotactile enhancement of cortical tracking of speech and impact on behavior

In A-only, we observed a significant reduction in both syllabic and phrasal CTS in the presence of the multi-talker background, with a stronger dampening in the right compared to the left hemisphere, consistent with previous studies (Destoky et al., 2019; Vander Ghinst et al., 2016, 2019). Under these adverse auditory conditions, congruent speech-derived vibrotactile stimulation significantly restored syllabic CTS at the right STAC. Given that CTS was measured using coherence, the present results may be interpreted as reflecting a tactile-driven facilitation of oscillatory activity in the STAC. More specifically, this non-auditory input may influence the excitability of auditory circuits, promoting stronger phase-locking to the low-frequency rhythms of the attended speech than in unimodal conditions (Giraud & Poeppel, 2012; Peelle & Sommers, 2015). Indeed, the impact of continuous tactile input on auditory processing has been revealed to act in a phase-dependent manner (Fu & Riecke, 2023; Jagt et al., 2024). An alternative proposal is that vibrotactile input modulates attentional state, which can also impact phase-locking at early stages of sensory (e.g., auditory) processing (Lakatos et al., 2009). Since CTS in multi-talker backgrounds is attention-dependent (Rimmele et al., 2015), further work is needed to distinguish which of these two mechanisms takes precedence. Moreover, coherence cannot fully resolve the neural mechanisms at the basis of CTS, determine whether increased rhythmic synchronization reflects oscillatory phase alignment or repeated stimulus-evoked responses, or exclude potential oscillatory power contributions.

The syllabic-level specificity of this audio-tactile effect may reflect intrinsic cortical processing constraints, as somatosensory cortices operate within shorter temporal integration windows than those required for effective integration of phrasal-level information (i.e., up to 1–2 seconds) (Romo & Rossi-Pool, 2020). This was confirmed here by the consistent presence of an S1 cortex source in ATc conditions for syllabic and word, but not phrasal, CTS (Fig. 4; Table 1). Alternatively, for phrasal CTS, the same amount of neuromagnetic data includes less oscillatory cycles than at syllabic rates, which may reduce sensitivity for detection of effects at such slower timescales. The right-hemispheric lateralization of this effect may further stem from (i) the established role of right auditory areas in syllabic-level processing and CTS (Abrams et al., 2008; Poeppel, 2003), (ii) robust audio-tactile integrative effects observed in the hemisphere contralateral to the side stimulated tactilely (left-hand stimulation in our study) (Lütkenhöner et al., 2002), and (iii) the right hemisphere’s stronger engagement in audio-tactile integration and multisensory processing (Guilleminot et al., 2023; Hoefer et al., 2013; Molholm et al., 2002). Nonetheless, whether this right-hemispheric CTS enhancement would be maintained even when providing vibrotactile stimulation to the right palm remains an open question.

Another key finding of the current study was that, in multi-talker conditions, the greater the syllabic CTS increase at right STAC in audio-tactile compared to the unimodal condition, the better the participants’ performance on questions related to the speech content. This brain-behavior association substantiates previous findings of a positive link between audio-tactile CTS and speech intelligibility (Riecke et al., 2019). The absence of a correlation between the CTS and behavioral benefit (i.e., ATc–A-only difference for both measures) suggests, however, that this association may not involve a close mapping between the improvement at the two levels, consistent with prior findings (Riecke et al., 2019). More generally, these results reinforce the role of syllabic CTS in supporting speech understanding in noisy environments, as demonstrated using theta-band vibrotactile input (Guilleminot & Reichenbach, 2022), transcranial current stimulation (Keshavarzi et al., 2020) and in clinical or developmental populations with reduced speech-in-noise ability (Bertels et al., 2023; Vander Ghinst et al., 2019, 2021). Taken together, these findings suggest that syllabic CTS may serve as the neural scaffold for audio-tactile comprehension enhancement in multi-talker conditions.

### 4.3 Comparison with visual enhancement

Visual speech (i.e., seeing the speaker’s articulatory facial gestures) did not significantly increase comprehension compared to A-only in either the quiet or multi-talker condition. At the neural level, CTS was additionally not enhanced in AV in any frequency band. Comparable absence of visual effects on coherence-based CTS at STAC/STG has been previously reported for vocoded speech presented in AV conditions (Aller et al., 2022). Moreover, intracranial STG recordings comparing word-related activity in AV and A-only conditions indicate a subregion- and context-dependence of these multisensory effects, with certain STG sites showing no robust AV-A differentiation (Ozker et al., 2017). The spatial leakage inherent to non-invasive source estimation in MEG may thus have constrained the ability to detect focal AV effects on CTS at STAC.

These results, however, diverge from prior studies reporting increased speech understanding and CTS in multi-talker conditions with visual speech support (Bertels et al., 2023; Destoky et al., 2020). From a methodological point of view, this might be attributable to the CTS metric used. These works, indeed, relied on stimulus reconstruction, which considers the *distributed* (i.e., whole-brain) multichannel activity for CTS computation. As such, it may be more sensitive to AV effects distributed across broader areas than our coherence analysis. Design factors may have also limited the AV CTS and behavioural benefits, including the employed SNR (i.e., 0 dB), which could have not been sufficient to maximize AV perceptual benefit (Crosse, Di Liberto, & Lalor, 2016), as well as the used visual stimulus material. Here, narrators exhibited minimal facial movement and expression, and such extraoral visual features of speech can impact AV benefit effects (Thomas & Jordan, 2004). Lastly, participants were not explicitly instructed to focus on the narrators’ lips, which are essential for encoding envelope information (Chandrasekaran et al., 2009). Insufficient visual attendance to the speaker’s lips (potentially further disrupted by the speakers’ eye movements due to teleprompter reading), may have limited the extraction of articulatory cues, thereby preventing speech envelope encoding in auditory cortices (Chandrasekaran et al., 2009; Peelle & Sommers, 2015).

In contrast, vibrotactile input in AT conditions was directly derived from amplitude envelope modulations. Thus, it may have provided a more robust transmission channel for envelope information than visual speech, in accordance with reports of only modest direct correlation between lip movements and speech envelope (Bourguignon et al., 2020). In addition, vibrotactile input can exert modulatory effects on neural activity even in the absence of measurable behavioural effects (Fu & Riecke, 2023; Riecke et al., 2019). As such, tactile speech cues may have had a more pronounced effect on CTS responses. For future studies investigating AV CTS enhancement, we underscore the importance of pilot testing for optimal SNR/task difficulty selection and of more careful gaze instruction or online monitoring (e.g., using eye-tracking).

### 4.4 Functional connectivity changes in ATc conditions

In the ATc multi-talker condition, we evidenced increased ipsilateral low-beta functional connectivity between the right STAC and the right AG and posterior ITG. The AG is a well-known convergence zone of multisensory input (Seghier, 2013), critical in binding cross-modal information for conceptual representation. Similarly, the right ITG is a high-level multimodal area involved in cross-modal expectation violation (Sherman et al., 2016). Given the purported role of beta-band oscillations in top-down predictive coding during speech processing (Hovsepyan et al., 2023), this increased synchronization may be associated with a top-down mechanism of contextual filtering (i.e., STAC auditory representations that mismatch the concurrent tactile input are suppressed selectively), potentially enhancing the accuracy of syllabic CTS. Furthermore, while lexico-semantic processing is typically left-lateralized, right-hemispheric AG and ITG have both been linked to such processes (Binder et al., 2009; Hickok & Poeppel, 2007; Seghier, 2013). Thus, increased STAC connectivity with these regions may accompany a more refined temporal resolution of speech envelope encoding at STAC through lexico-semantic priors, using the perceived audio-tactile speech cues to constrain linguistic predictions. Given the absence of left-hemispheric involvement, however, this interpretation warrants further investigation, for example through manipulations of tactile laterality and the lexico-semantic predictability of the speech input.

Decreased alpha-band functional connectivity between the right STAC and the right precuneus was also observed in the multi-talker ATc condition (vs. A-only). The precuneus, a key node of the default-mode network (DMN), is involved in internally-oriented processing (Utevsky et al., 2014), while alpha oscillations play a key role in mediating DMN functional connectivity (Clancy et al., 2022) and disengagement from the sensory environment (Foxe & Snyder, 2011). This decrease might thus reflect increased externally-oriented processing in the ATc condition, which may be associated with improvements in CTS.

These findings highlight a broader, long-range network-level mechanism during ATc, where higher-order, multisensory areas exhibit functional connectivity changes with STAC, which could potentially be consistent with refined speech envelope tracking through beta-mediated predictive feedback and alpha-mediated attentional shifts. This underscores the engagement of associative cortical regions—rather than direct somatosensory-auditory connections—under adverse auditory conditions with audio-tactile multisensory input.

### 4.5 CTS in unimodal, non-auditory conditions

Despite the lack of statistically significant AV condition-related effects on comprehension or CTS values, we uncovered a phrasal CTS source at the right posterior STG during the V-only condition. This corresponds to previous findings (Bourguignon et al., 2020) showing that visual articulatory cues can drive the synthesis of slow (<1 Hz) speech temporal envelope cues in auditory cortices through an analysis-by-synthesis mechanism (Poeppel & Monahan, 2011). Notably, however, we failed to replicate the emergence of V-only syllabic CTS in occipital areas, and of word CTS in left temporal areas (Bourguignon et al., 2020). In that study, V-only CTS of unheard speech at word and syllabic levels was directly linked to attendance to mouth movements, as it was abolished when mouth movements were controlled for through partial coherence analyses. The absence of similar V-only effects in our data may therefore indicate that participants were not consistently attending to the lip area, which could help explain the null AV effects. The emergence of phrasal CTS near STAC in the absence of such syllabic/word CTS in V-only conditions points to a distinct mechanism occurring at two timescales: phrasal rhythms may be inferred from higher-level predictive processes, despite limited visual focus to lips, whereas syllabic/word-level CTS rely on detailed attention to visual lip movements.

In the T-only condition, for all CTS frequencies, we found that significant CTS local maxima also localized to the right superior temporal/STG area (adjacent to STAC), in agreement with the extensive work on the somatosensory modulation of auditory and peri-auditory activity (Caetano & Jousmäki, 2006; Fu et al., 2003; Hackett et al., 2007; Lakatos et al., 2007). The ability of a continuous, speech-derived vibrotactile input to entrain auditory activity even in the absence of auditory input further strengthens the perspective of phase-based accounts of multisensory contributions to speech perception (Peelle & Davis, 2012; Peelle & Sommers, 2015). In the T-only condition, we also uncovered that activity at right S1 cortex was entrained by the speech-derived vibrotactile stimulation at word and syllabic frequencies, likely reflecting the direct processing of the vibrotactile input. The additional syllabic CTS source in the right premotor cortex may be linked to the motor theory of speech perception (Peelle et al., 2022), whereby premotor/motor regions are recruited to enhance speech perception. However, its exclusive presence in T-only and not in AT conditions suggests it may instead be a direct by-product of the tactile tracking of the speech-derived vibration in the absence of concurrent auditory speech, potentially engaging more widespread somatosensory responses.

### 4.6 Limitations

The uncovered vibrotactile syllabic CTS enhancement is suggestive of an integrative mechanism, whereby neural populations in the STAC exhibit a stronger response in ATc relative to the response to unisensory stimuli (auditory speech and speech-derived vibrations) alone. A limitation of the current study is that we cannot fully resolve whether this effect’s underlying computation is additive (i.e., a linear summation of unimodal responses; AT = A-only + T-only) or super-additive (i.e., the summation exceeding the linear sum of unimodal responses; AT > A-only + T-only) (Meredith & Stein, 1986). We did not find evidence of super-additive effects, consistent with previous work on audio-tactile speech (Schulte et al., 2025), although evidence in support also exists (Riecke et al., 2019). Coherence, however, is a bounded, non-linear and positively biased measure. Its use for additive predictions (e.g., A-only + T-only) can thus artificially inflate the obtained sum, concealing any potential super-additive effects. Hence, we caution against conclusions regarding super-additivity (or lack thereof) using coherence metrics alone. Alternative CTS measurements, such as cross-validated encoding/decoding models, could potentially better uncover the precise nature of these audio-tactile multisensory effects.

With respect to functional connectivity changes associated with the ATc condition, the effects reached statistical significance but were near-threshold and uncorrected across frequency bands comparisons. These findings thus warrant future investigation to establish their reliability and functional significance. Moreover, further replications are necessary to determine whether neighboring sources’ activity may have contributed to the observed connectivity patterns. Importantly, these connectivity findings are correlational and cannot solely establish the causal role of the uncovered areas in the ATc-related CTS increase. The involvement of the extra-auditory areas shown here to have stronger connectivity with STAC in ATc (i.e., ITG, AG) may be explored by, for example, testing whether their transient disruption (through transcranial or alternative current stimulation) will reduce the ATc-related CTS increase. Such causal manipulations would provide stronger evidence that the observed connectivity patterns truly reflect functionally relevant top-down influences on CTS.

An added limitation of this work is the use of yes/no content questions for the comprehension evaluation. While suitable for the employed material (i.e., connected speech), these lack granularity and are more susceptible to guessing than other comprehension measures. The one-to-one correspondence between each question and a single condition block (i.e., video segment) further constrains this granularity. Including multiple content questions per block and presenting them directly after each block rather than at the end of the video could have improved behavioral measurement accuracy, despite more frequent task-switching. These factors may have contributed to the reduced sensitivity to detect significant behavioural AV effects, widely documented previously. Future studies could complement this evaluation with more complex, discourse-level comprehension tasks (e.g., free recall or paraphrasing). Alternatively, paradigms allowing for more precise speech perception measures, such as sentence recognition thresholds, would provide a more direct assessment of speech perception and strengthen CTS-behavior associations, as they are less prone to memory demands or attentional effects. Optimally, to dissociate perceptual from high-level processing, both discourse- and sentence-level measurements would be assessed within the same participants.

### 4.7 Conclusion

This study demonstrates that, in a multi-talker scenario, supplemental, congruent speech-derived vibrotactile stimulation enhances comprehension of an attended speaker and gives rise to two distinct neural manifestations: improved temporal envelope encoding at the right auditory cortex (contralateral to the stimulated side) and changes in functional connectivity between this auditory area and ipsilateral, higher-order multisensory regions. These findings shed light on the neural pathways involved in audio-tactile speech enhancement, highlighting the potential of speech-derived vibrotactile stimulation to modulate speech-related neural activity toward improved speech comprehension in adverse auditory conditions.

## Supplementary Material

Supplementary Material

## Data Availability

Data and code supporting the current results will be provided upon reasonable request to the corresponding author and after the approval of the institutional authorities (Hôpital Universitaire de Bruxelles and Université libre de Bruxelles).
