# Supplementary material for: Can you feel what I am saying? Speech-based vibrotactile stimulation enhances the cortical tracking of attended speech in a multi-talker background
Source: Imaging Neurosci (Camb). 2026 Jul 21;4:IMAG.a.1305. doi: 10.1162/IMAG.a.1305 (PMC13389744; doi:10.1162/IMAG.a.1305)
Supplement: Supplementary Material [file IMAG.a.1305_supp.pdf]

Supporting Information

Section 4.2.4: Final condition orders

Order 1: multi-talker A-only, T-only, quiet AV, multi-talker ATi, V-only, quiet ATc, multi-talker AV, quiet A-only, multi-talker ATc;

Order 2: multi-talker ATc, quiet AV, multi-talker A-only, T-only, quiet A-only, multi-talker ATi, V-only, quiet ATc, multi-talker AV;

Order 3: multi-talker ATi, quiet AV, multi-talker A-only, quiet ATc, multi-talker AV, T-only, quiet A-only, multi-talker ATc, V-only;

Order 4: multi-talker AV, quiet A-only, multi-talker ATc, quiet AV, multi-talker ATi, V-only, quiet ATc, multi-talker A-only, T-only).
